# Health-related quality of life and its predictors among epilepsy patients in Ethiopia: Systematic review and meta-analysis

**DOI:** 10.1371/journal.pone.0324363

**Published:** 2025-06-03

**Authors:** Chilot Kassa Mekonnen, Hailemichael Kindie Abate, Abere Woretaw Azagew, Muluken Chanie Agimas

**Affiliations:** 1 Department of Medical Nursing, School of Nursing, University of Gondar, Gondar, Ethiopia; 2 Department of Epidemiology and Biostatics, Institute of Public Health, University of Gondar, Gondar, Ethiopia; University of Tabuk, SAUDI ARABIA

## Abstract

**Introduction:**

Epilepsy is a common non-communicable neurological disorder associated with recurrent seeding of cerebral neurons or brain cells and episodes of unprovoked seizures with or without loss of consciousness. Although there are studies on the health-related quality of life of epilepsy patients in Ethiopia, there are remarkable variations in the estimates of health-related quality of life.

**Objectives:**

This systematic review and meta-analysis aimed to determine the pooled effect size of the health-related quality of life of adult epilepsy patients in Ethiopia.

**Methods:**

Original articles about the health-related quality of life among epilepsy patients in Ethiopia were searched through known and international databases (PubMed, Scopus, and Web of Science) and search engines (Google and Google Scholar). Data were extracted using a standard data extraction checklist developed according to Joanna Briggs Institute (JBI). The I^2^ statistics were used to identify heterogeneity across studies. Funnel plot asymmetry and Egger’s tests were used to check for publication bias. The STATA version 11 software was employed for statistical analysis to pool the mean scores of health-related quality-of-life.

**Result:**

A total of 16 cross-sectional studies with a sample size of 5294 took part. The pooled overall mean score of health-related quality of life among epilepsy patients in Ethiopia was 52.82 ± 13.24 [95%CI (46.41, 59.21)], I2 = 100%, p-value <0.001. Besides, the overall pooled mean score of health-related quality of life based on the measuring tools WHO-QOL-BREF, QOLIE-31, and QOLIE-10 was 51.50 ± 11.32 (49.11, 53.90), 59.99 ± 12.67 (52.22, 67.77), and 44.33 ± 21.23 (13.09, 75.57) respectively.

**Conclusion:**

The least overall pooled mean score of HRQOL was the QOLIE-10 measuring tool epileptic patients’ mean score compared to other health-related quality-of-life measuring tools such as WHOQOL-BREF, and QOLIE-31. Moreover, the least overall pooled mean score of HRQOL using the WHOQOL-BREF was the environmental domain followed by the emotional domain, as compared to all other domains in each measuring tool of health-related quality of life.

## Introduction

Epilepsy is defined as a common non-communicable neurological disorder associated with recurrent seeding of cerebral neurons or brain cells and episodes of unprovoked seizures with or without loss of consciousness [1]. However, people who have had only seizures related to other well-defined febrile conditions are not considered as epilepsy [2]. Health-related quality of life (HRQOL) is a complex and multidimensional conception which encompasses physical health, psychological state, social, and environmental maladjustments [3]. Studies conducted on health-related quality of life of epilepsy patients become increasingly common in low and middle-income countries, particularly in Ethiopia. However, there are remarkable variations in the estimates of health-related quality of life associated with measurement differences and reporting [4–6].

In Sub-Saharan Africa, a prevalence of 9.39 per 100 epileptic cases was found [7]. Furthermore, the global prevalence of epilepsy is reported that there are over 50 million individuals suffering from epilepsy and of these more than 125,000 die each year [8]. According to the Global Burden of Epilepsy Report, nearly 13 million adjusted life years disabilities occur due to epilepsy annually [9]. Evidence in the US revealed that the prevalence of idiopathic epilepsy declined from 76.9% to 75.1 between 1999 and 2017 [10]. Epilepsy has a remarkable impact on patient’s well-being, social capital and health-related quality of life due to various contributing factors such as medication adherence, socio-economic cost, decreased employment probability, and comparatively low income [11].

Moreover, individuals with epilepsy are prone to social isolation or stigma related to decreased self-esteem, comorbid depression, anxiety, and suicidal attempts as a result of predicting recurrent seizure episodes which decline the health-related quality of life of patients [12]. Epilepsy patients have physical, psychological, cognitive, social, and environmental maladjustments which result in a significant loss of health-related quality of life [13]. There are inconsistent findings in the health-related quality of life mean scores among studies conducted in Ethiopia. For instance, from the lowest mean score of 19.85 ± 6.91 to the highest 79.14 ± 25.46. Therefore, this systematic review and meta-analysis tried to minimize the variation by generating the pooled estimates of individual studies.

## Materials and methods

### Registration and report of the study protocol

This systematic review and meta-analysis have utilized the guidelines developed by the Joanna Briggs Institute (JBI) for Systematic Reviews [14] and the report is written consistent with the revised 2020 PRISMA guidelines [15]. This systematic review and meta-analysis title and its protocol have been registered in the PROSPERO online database (with registration number CRD420234880041). The result of this review presentation was consistent with the standard preferred Reporting Items [15] for the Systematic Review and Meta-analysis (PRISMA) checklist (S1 File).

### Searching strategies

The known and international databases (PubMed, Scopus, Web of Science, and Cochran Library) and search engines (google and Google Scholar) were used to locate research articles on health-related quality of life of epilepsy patients in Ethiopia. The string for searching was developed using “AND” and “OR” Boolean operators with the keywords extracted from the Medical Subject Headings (MeSH) database. The search strategy was based on the research question of this review and utilized the **CoCoPop (Co = **Condition**, Co = **Context**, Pop = **Population**) model.** The article locating strategy was through “health-related life quality “**OR** HRQOL **OR** “epilepsy patients HRQOL” **OR** “quality of life” **OR** “epilepsy patient* HRQOL” **AND** “epilepsy patient*” **OR** “adult epilepsy patients” **OR** “Seizure disorder*” **OR** *Seizure **AND** *Ethiopia **OR** Ethiopia. This search strategy primarily aimed to trace all reviewed (published) and unpublished primary studies. The list of all retrieved primary articles and systematic review and meta-analysis references were also screened or cross-referenced to get extra studies. The sources of information range from electronic databases to direct contact with the principal investigator if the need arises. The first search through Pub Med, Cochran Library, Scopus, Web of Science, Google, and Google Scholar was done in November 2023. The final search for updating was conducted from November 20/ 2023 to December 20/ 12/2023. The publication date was used as a filter mechanism in which articles published from January 2014 to December 2023 were included in the current systematic Review and Meta-analysis study to generate the most recent evidence for the scientific community.

### Eligibility criteria

In this review the primary articles were eligible if and only if (1) they reported epilepsy patients (2) aged≥ 18 years old (3) those articles reported the overall mean score of health-related quality of life (4) observational studies (both analytical and descriptive cross-sectional) (5) articles published in English and only from Ethiopia were included, and (6) published from 2014 to 2023 were included in this particular systematic review and Meta-analysis. Nonetheless, after a thorough examination of the titles and abstracts using eligibility criteria, irrelevant studies such as conference reports, qualitative papers, retracted articles and published in languages other than English were excluded. In this systematic review and meta-analysis time and language restriction was used just to ensure the study remains focused, up-to-date, feasible, and relevant to the scientific community. Although there is no rough and fixed rule in the publication time of articles to be included in systematic review and meta-analysis, we thought restricting the period to 10 years of publication help us to minimize bias from outdated studies. It is recommended by the scholars to include articles published within ten years which helps to focus on the most recent and relevant evidence as research and clinical practice evolve [16]. Furthermore, language restrictions sought from feasibility, time and resource constraints, access to studies, and publication bias perspectives. However, that was not the case in our study as far as all articles in Ethiopia published in English as eligible for the final analysis.

### The study selection and outcome

After comprehensive searching, all located citations were selected and exported to Endnote citation manager software version X7. Following this, irrelevant and duplicated articles were removed. Then three independent researchers (HKA, MCA and AWA) screened each particular article for its title, abstract, and full text by far and cross-checked it against the inclusion criteria. The other research team (CKM, MCA, and AWA) checked the screened articles with full text for details by already defined criteria to take it to the final review process. Any sort of disagreement between the research team while including and excluding articles on predefined criteria of this particular review was resolved by a thorough discussion of the team. The exclusion of the articles was presented with countable reasons which could be consistent with the pre-defined criteria. The result of searching further screening and inclusion process of articles in this review was done in agreement with the PRISMA guidelines for Systematic Review and Meta-analysis 2020.

### Quality appraisal of included studies

The methodological quality of the included studies was critically appraised by three independent reviewers (AWA, CKM and HKA) by using the standardized JBI critical appraisal tool for the studies reporting prevalence [14]. This critical appraisal tool consists of 9 items designed to oversee the study population, sample size adequacy of the study, study subject and setting, reliability of condition or problem measurement, the appropriateness of the Statistical test used to analyze the data, and adequacy of the response rate of each selected article for the review process (**Table 1**). Each item has an answer of “No”, “Yes” or “unclear” to rate its risk of bias (ROB). After the critical appraisal, the reviewers decided to include or exclude screened articles based on the overall quality of the appraisal score in which those articles with the lowest score out of 9 were considered as poor quality or high risk of bias. The article was prone to exclude when the score was below average that is 4.5 of the three independent reviewers. In this regard, there had to be more than 3 “No” or unclear” quality categories for the article to be excluded from the review. This critical appraisal threshold was supported by a previously published systematic review and meta-analysis study [17]. Any sort of disagreement between the involved reviewers was solved through the discussion of the reviewers. Furthermore, if the disagreement unfolds, the fourth reviewer was indicated to oversee the source of the doubt and reach to consensus.

**Table 1:** Methodological Quality assessment of the included primary studies.

Author	Q1	Q2	Q3	Q4	Q5	Q6	Q7	Q8	Q9	Score/9
Abadiga et al. [25]	Y	Y	Y	Y	Y	Y	Y	N	Y	8
Tefera, G. M. [31]	Y	Y	N	Y	Y	Y	Y	Y	Y	8
Tegegne, M.T. [32]	Y	Y	N	Y	Y	Y	Y	Y	Y	8
Minwuyelet F, et al. [24]	Y	Y	N	Y	Y	Y	Y	N	Y	8
Stotaw et al. [30]	Y	Y	N	Y	Y	Y	Y	N	Y	8
Addis, B., et al. [21]	Y	Y	Y	Y	Y	Y	Y	Y	Y	9
Gebre, A.K., et al. [27]	Y	Y	Y	Y	N	Y	N	Y	Y	8
Minyihun,A.,et al. [23]	Y	Y	N	Y	Y	Y	N	Y	Y	7
Guday, E., et al. [19]	Y	Y	Y	Y	Y	Y	Y	Y	Y	9
Kassie AM, et al. [22]	Y	Y	Y	N	Y	Y	Y	Y	UN	7
Mesafint et al. [4]	Y	Y	Y	Y	Y	Y	Y	Y	UN	8
Muche, E.A., et al. [29]	Y	Y	N	Y	Y	Y	Y	Y	Y	8
Tsigebrhan, R., et al. [33]	Y	Y	N	Y	Y	Y	Y	Y	Y	8
Wudu Yesuf. [20]	Y	Y	Y	Y	Y	Y	Y	Y	Y	9
Hailu, D.S.E. [28]	Y	Y	Y	Y	N	Y	Y	Y	Y	8
Alemu, A., et al. [26]	Y	Y	Y	Y	Y	Y	Y	Y	Y	9

N = No, NA = Not Applicable, UN = Unclear, Y = Yes,

Q1: Was the sample frame appropriate to address the target population? Q2: Were study participants sampled appropriately? Q3: Was the sample size adequate? Q4: Were the study subjects and the setting described in detail? Q5: Was the data analysis conducted with sufficient coverage of the identified sample? Q6: Were valid methods used for the identification of the condition? Q7: Was the condition measured in a standard, reliable way for all participants? Q8: Was there an appropriate statistical analysis? Q9: Was the response rate adequate and if not, was the low response rate managed appropriately?

### Data extraction

Data were independently extracted by five authors using a standardized data extraction format that was developed according to the 2014 JBI Reviewers’ Manual [18]. The tool includes Authors, Region, study year, study design, sample size, the mean score of health-related quality of life of epilepsy patients, a tool used to measure the outcome, response rate, and risk of bias assessment score included in the extraction. The data were extracted by three ((HKA, MCA and AWA) independent reviewers and any inconsistent data were cross-checked (S2 File). The disagreement between the reviewers was solved by a thorough discussion.

### Data synthesis and analysis

The outcome of included primary studies was narratively presented and expanded with supplementary materials in text, tables, and figures where necessary. All necessary and relevant information from every article was extracted through a Microsoft Excel spreadsheet, and exported to STATA Version 11 for further analysis. The random-effects model was employed to estimate the pooled effect size of health-related quality of life of epilepsy patients due to the presence of heterogeneity. Heterogeneity was identified by using the standard chi-square and I-square statistical tests. The variation between different study characteristics such as the region where the primary article was conducted, outcome ascertainment tool, and year of publication were investigated through subgroup analyses. This subgroup analysis could demonstrate the sources of heterogeneity and let the researcher for another remedy such as the use of meta-regression to treat this heterogeneity. Moreover, the publication bias was assessed through visual inspection of the funnel plot, Begg- Mazumdar Rank correlation tests and Egger’s test to see the funnel plot’s asymmetry. The influence of individual articles on the overall pooled effect size or the overall mean score of HRQOL was assessed by using sensitivity analysis. The forest plot with 95% CI was used to present the overall pooled mean score as well as the subgroup pooled overall mean score of HRQOL among epilepsy patients in Ethiopia. Moreover, the bivariate and multivariate meta-regression analysis was conducted to identify the potential covariates that brought about the variations.

## Results

### The article selection and outcome

**Fig 1:**
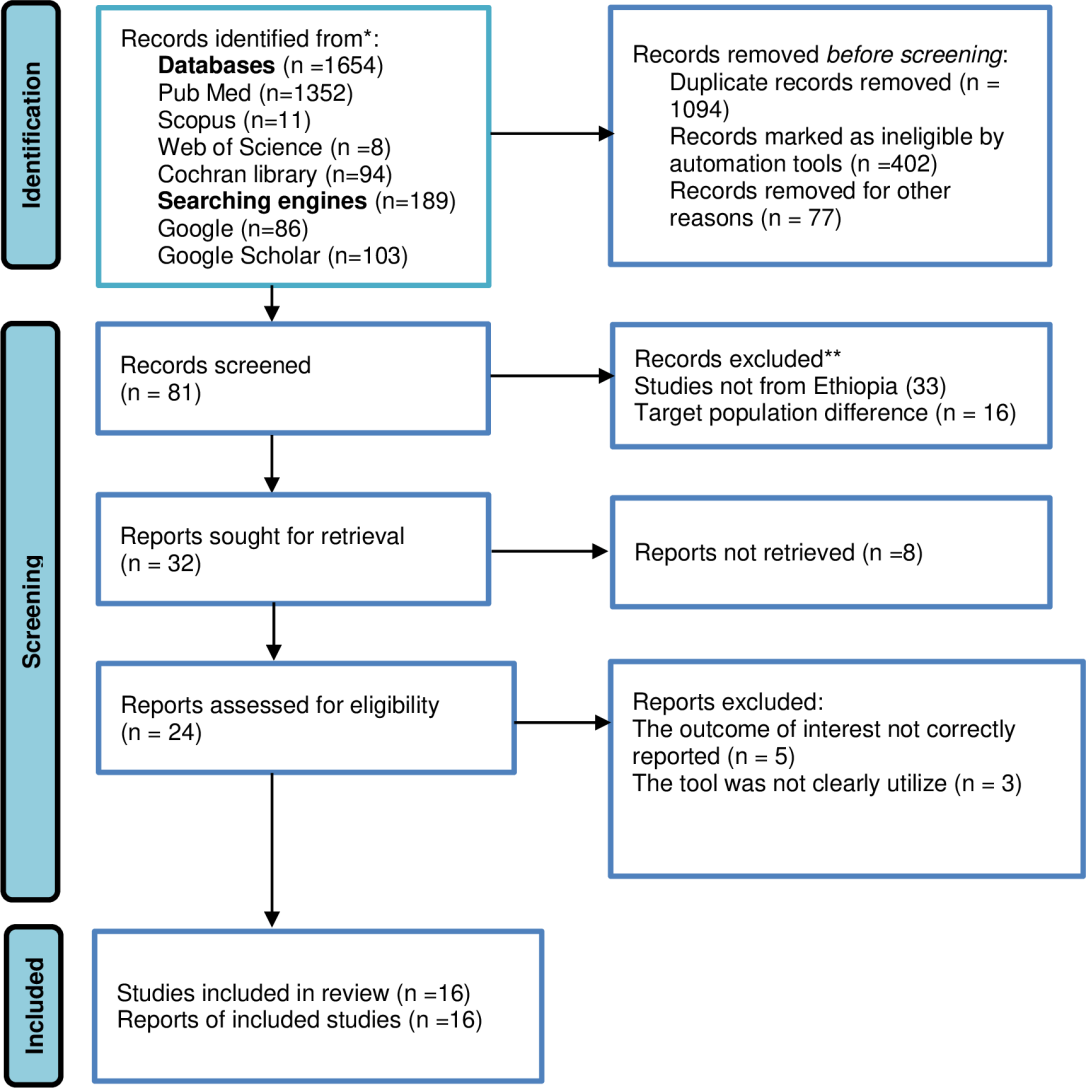
PRISMA flow diagram of the included studies.

In this systematic review and meta-analysis study, a total of (1654) articles related to the quality of life of epilepsy patients in Ethiopia were identified using electronic databases and search engine websites. Among overall articles, 1094 were removed for being irrelevant and duplicated. The other pretty sizable articles were removed for not being ineligible (study design and Title difference) by using the Endnote automation tool and other reasons (402 vs. 77) respectively. The remaining 81 articles were eligible for screening. Of these screened 49 papers were excluded due to the country of study or not being conducted in Ethiopia and target population difference (those articles conducted among children). With further screening, 32 articles were sought for retrieval and 8 were not retrieved for one and the other reason. Moreover, 24 research articles were assessed for eligibility to be included in the review process, but with the outcome of interest and measurement tool ambiguity a total of 8 articles were excluded. Finally, 16 original research articles were incorporated into the systematic review and meta-analysis (**Fig 1**).

### The methodological quality assessment of included studies

There are a total of 16 articles assessed for methodological quality using a 9-point score tool developed by JBI for observational prevalence studies. The outcome of the quality appraisal ranged from moderate to high methodological quality in which three studies [19–21] scored 9 points, two studies. [22,23] scored 7 points, and the remaining eleven studies [4,24–32] scored 8 points (**Table 1**).

### Characteristics of the included primary studies

In this systematic review and meta-analysis, 5294, participants were included with a response rate of 100%. The studies included in this review were observational cross-sectional studies published from 2014 to 2023. The smallest sample size was 78 from the study conducted in the Amhara region, Ethiopia [23] followed by 121 from the Oromia, region of Ethiopia [31]. Whereas, the largest sample was 462 from Amhara region, Ethiopia [19] followed by 439 the study from Addis Ababa, Ethiopia [4]. Moreover, eight studies [4,19,21,24–26,30,32] used WHOQOL-BREF measuring, six studies [20–22,24,27,28] used QOLIE-31, and the rest two studies [29,33] used QOLIE-10 measuring tool to measure health-related quality of life of epilepsy patients. Besides, the statistical models employed were linear regression and logistic regression in which four studies [24,26,30,32] used logistic regression and the remaining twelve studies used linear regression (**Table 2**).

**Table 2:** Characteristics of the included primary studies in the review.

Author/reference	Publication year	Region	Study design	Sample size	Measuring tool	Statistical model
Abadiga et al. [25]	2019	Oromia, Ethiopia	Crossectional	392	WHOQOL-BREF	Linear regression
Tefera, G. M. [31]	2020	Oromia, Ethiopia	Crossectional	121	WHOQOL-BREF	Linear regression
Tegegne, M.T. [32]	2014	Oromia, Ethiopia	Crossectional	415	WHOQOL-BREF	Logistic regression
Minwuyelet F, et al. [24]	2022	Amhara, Ethiopia	Crossectional	402	WHOQOL-BREF	Logistic regression
Stotaw et al. [30]	2022	Amhara, Ethiopia	Crossectional	384	WHOQOL-BREF	Logistic regression
Addis, B., et al. [21]	2021	Amhara, Ethiopia	Crossectional	370	QOLIE-31	Linear regression
Gebre, A.K., et al. [27]	2018	Tigray, Ethiopia	Crossectional	175	QOLIE-31	Linear regression
Minyihun,A.,et al. [23]	2022	Amhara, Ethiopia	Crossectional	78	QOLIE-31	Linear regression
Guday, E., et al. [19]	2022	Amhara, Ethiopia	Crossectional	462	WHOQOL-BREF	Linear regression
Kassie AM, et al. [22]	2021	Amhara, Ethiopia	Crossectional	395	QOLIE-31	Linear regression
Mesafint et al. [4]	2020	Addis Ababa, Ethiopia	Crossectional	439	WHOQOL-BREF	Linear regression
Muche, E.A., et al. [29]	2020	Amhara, Ethiopia	Crossectional	354	QOLIE-10	Linear regression
Tsigebrhan, R., et al. [33]	2021	Southern, Ethiopia	Crossectional	237	QOLIE-10	Linear regression
Wudu Yesuf. [20]	2019	Oromia, Ethiopia	Crossectional	340	QOLIE-31	Linear regression
Hailu, D.S.E. [28]	2018	Oromia, Ethiopia	Crossectional	304	QOLIE-31	Linear regression
Alemu, A., et al. [26]	2023	Southern, Ethiopia	Crossectional	423	WHOQOL-BREF	Logistic regression

### Health-related quality of life of epilepsy patients in Ethiopia

**Fig 2:**
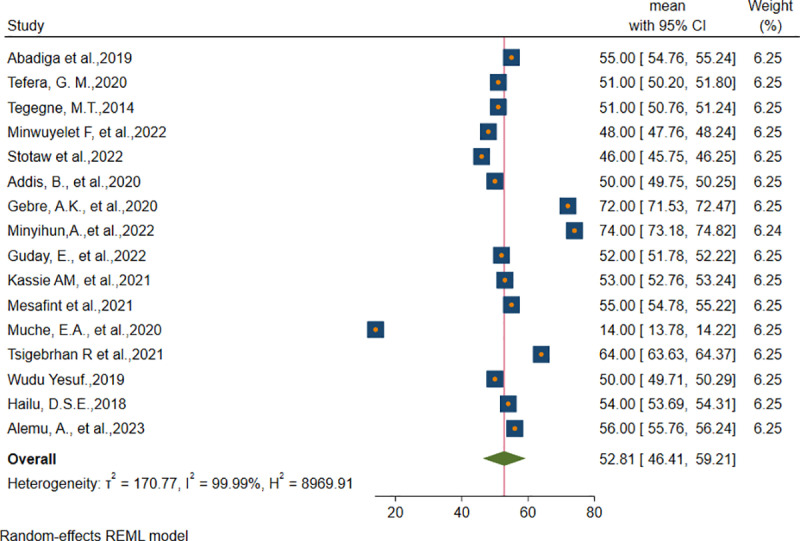
Pooled overall mean score of Health-related quality of life of epilepsy patients in Ethiopia.

A total of 16 primary articles were appraised and retrieved to pool the overall mean score of health-related quality of life among epilepsy patients in Ethiopia. All the included studies used raw data transformation to be in the scale range of 0–100. The mean score of health-related quality of life overall score of individual studies ranged from 19.85 ± 6.91 to 79.14 ± 25.46 in the Amhara Region, Ethiopia respectively. The pooled overall means score of health-related quality of life among epilepsy patients in Ethiopia was 52.82 ± 13.24 [95%CI (46.41, 59.21)], **I**^**2 **^**= 99.9%, p-value <0.001.** The effect size of the overall pooled mean score of health-related quality of life among epilepsy patients in Ethiopia was presented using a forest plot (**Fig 2).**

### WHO-QOL-BREF, QOLIE-31, and QOLIE-10 domain-based overall mean scores of health-related quality of life

The pooled mean score of health-related quality of life-based on the measuring tools was 51.50 ± 11.32(49.11, 53.90) for WHO-QOL-BREF, 59.99 ± 12.67 (52.22,67.77) for QOLIE-31, and 44.33 ± 21.23 (13.09, 75.57) for QOLIE-10respectively. Hence, WHOQOL-BERF has four domains of health such as physical domain of health, psychological domain of health, Social domain of health, and environmental domain of health. Whereas, the domain based pooled mean-score of WHO-QOL-BREF was physical 52.67 ± 16.35 [95%CI(40.70, 64.64)], I^2 ^= 96.3%, p-value <0.001, psychological 50.50 ± 20.04 [95%CI(39.26, 65.73)], I^2 ^= 100%, p-value <0.001, emotional 49.97 ± 13.78 [95%CI(32.34, 67.61), I^2 ^= 100%, p-value <0.001, and environmental 48.09 ± 21.14 [95%CI(38.31, 57.87)], I^2 ^= 100%, p-value <0.001 respectively. The lowest and highest mean scores were in the environmental and physical health domains respectively. Regarding, the QOLIE-31 measuring tool, it has seven domains with the highest and lowest mean scores of health-related quality of life 69.86 ± 23.06 [95%CI (62.99, 76.73)] in the domain of social functioning and 62.14 ± 18.26 [95%CI (57.11, 67.17)] in the overall quality of domain respectively (Table 3).

**Table 3:** Meta-analysis summary results for each domain of WHO-QOL-BREF, QOL-31, and QOLIE-10 health-related quality-of-life measuring tools among epilepsy patients in Ethiopia.

Variables	Domains	Pooled mean/ ʙ with 95%CI	Egger’s test	I-Square (%)	Regression Model
WHO-QOL-BREF	Physical health	52.67 ± 16.35(40.70, 64.64)	0.657	100	Random
	Psychological health	50.50 ± 21.04 (39.26, 65.73)	0.326	100	Random
	Emotional relation	49.97 ± 13.78 (32.34, 67.61)	0.093	100	Random
	Environmental Health	48.09 ± 21.14 (38.31, 57.87)	0.564	100	Random
	Overall mean	51.50 ± 11.32(49.11,53.90)	–	37.4	Random
QOLIE-31	Seizure worry	63.93 ± 16.94(54.26,73.59)	0.519	100	Random
	Overall QOL	62.14 ± 18.26(57.11,67.17)	0.001	31.12	Random
	Emotional wellbeing	63.06 ± 17.68(56.35, 69.77)	0.317	100	Random
	Energy/fatigue	67.90 ± 21.32(59.57, 76.23)	0.155	100	Random
	Cognitive function	67.90 ± 20.11(59.57, 76.23)	0.001	51.81	Random
	Medication effects	68.11 ± 18.76(60.13, 76.09)	0.659	100	Random
	Social functioning	69.86 ± 23.06(62.99, 76.73)	0.861	100	Random
	Overall pooled mean	59.99 ± 12.67 (52.22,67.77)		100	Random
QOLIE-10	Overall pooled mean	44.33 ± 21.23 (13.09, 75.57)	–	100	Random

### Subgroup analysis

In this review, the subgroup analysis was employed by using the country and the outcome ascertainment tool used in the primary reviewed articles. In the subgroup analysis by region the lowest pooled overall mean-score HRQOL using the random-effects model was 48.14 ± 21.06 [95%CI (36.14, 60.14), I^2^ = 100%, p < 0.001] in Amhara, Ethiopia followed by 52.21 ± 15.98 [95%CI (50.11, 54.30), I^2 ^= 99.6%, p < 0.001] in Oromia, Ethiopia.

Whereas the highest pooled overall mean-score HRQOL was 72.0 ± 19.63 [95%CI (71.53, 72.47), I^2^ = 0.00%, p < 0.001] in Tigray, Ethiopia followed by 60.0 ± 17.03 [95%CI (52.16,67.84), I^2^ = 99.9%, p < 0.0001] in Southern, Ethiopia. Moreover, in the subgroup analysis using the outcome measuring tool the highest pooled overall mean-score HRQOL using the random-effects model was 59.99 ± 12.67 [95%CI (52.22, 67.77), I^2^ = 1000%, p < 0.001] with outcome measuring tool of QOLIE-31 followed by 51.50 ± 11.32 [95%CI (49.11, 53.90), I^2^ = 37.4%, p < 0.022] using WHOQOL-BREF measuring tool of health-related quality of life among epilepsy patients in Ethiopia. Furthermore, in the subgroup analysis using publication year, a higher pooled overall mean-score HRQOL was 55.99 ± 17.43 [95%CI (52.25, 59.72), I^2^ = 86.5%, p < 0.001] using the publication year of ≥ 2021 (Table 4).

**Table 4:** Sub-group analysis of health-related quality of life of epilepsy patients using region, outcome measuring tool, and year of publication in Ethiopia.

Sub-group by	Number of articles	Sample size	Pooled mean score	I-square (%)	P-value
**Region**					
Oromia, Ethiopia	5	1572	52.21 ± 15.98 (50.11,54.30)	99.6	<0.01
Amhara, Ethiopia	7	2445	48.14 ± 21.06(36.14, 60.14)	100	<0.001
South, Ethiopia	2	660	60.0 ± 17.03(52.16, 67.84)	99.9	<0.001
Tigray, Ethiopia	1	175	72.0 ± 19.63(71.53, 72.47)	0.0	<0.001
Addis Ababa, Ethiopia	1	439	55.0 ± 14.87(54.78,55.22)	0.0	<0.001
**Measuring tool**					
WHOQOL-BREF	8	3041	51.50 ± 11.32(49.11,53.90)	99.9	<0.22
QOLIE-31	6	1662	59.99 ± 12.67(52.22,67.77)	100	<0.001
QOLIE-10	2	591	44.33 ± 21.23 (13.09, 75.57)	100	<0.001
**Year of publication**					
≤2020	8	2471	49.62 ± 20.18(37.04,62.21)	100%	<0.001
≥2021	8	2823	55.99 ± 17.43(52.25, 59.72)	91.32.%	<0.001

### Heterogeneity assessment

In this systematic review and meta-analysis, the analysis output using a random-effects model showed high variability across the primary articles included in the study (I^2^ = 96.3%, P < 0.001). This variability is inevitable in meta-analysis studies resulting from quality differences of the included studies, methodological differences, sample size, inclusion and exclusion, and the difference in measuring tools to ascertain the outcome of interest. Therefore, we have conducted the meta-regression analysis by using publication year, sample size, and standard error as covariates to figure out the potential source of heterogeneity among included studies. In this regard, the meta-regression analysis revealed that no significant correlation was found between the outcome of interest (HRQOL) and the included covariates by far (p = 0.997, for publication year and P = 0.932 for sample size). Hence, there was no statistically significant association and possible existence of variability as shown (Table 5). This again implies that the source of high variability (heterogeneity) could be due to chance or the other variables not investigated in this particular review. We have also conducted a baujate plot (S1 Fig) to identify outliers and a bubbles plot (S2 Fig) to identify the sources of heterogeneity.

**Table 5:** Meta-regression analysis to see between studies variation (heterogeneity).

Sources of heterogeneity	Coefficients	Std. Err	P-value
Publication year	.001369	.276529	0.798
Sample size	-.0015652	.0068149	0.982

### Publication bias assessment

**Fig 3:**
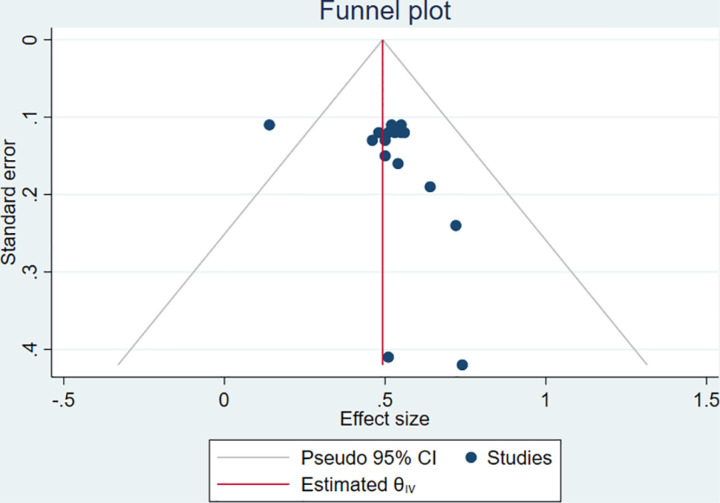
Funnel plot to assess publication bias.

In this particular systematic review and meta-analysis study publication bias was assessed by using visual inspection of a funnel plot which referred to asymmetry, as seven studies lay to the left and 9 studies were to the right of the line (Fig 3). Although it was not statistically significant as shown in Egger’s test (P = 0.068) which depicted that the outputs were not influenced by publication bias by one and the other reasons. It is evidenced that the asymmetry of the funnel plot is not always related to publication bias [34].

### Sensitivity analysis

**Fig 4:**
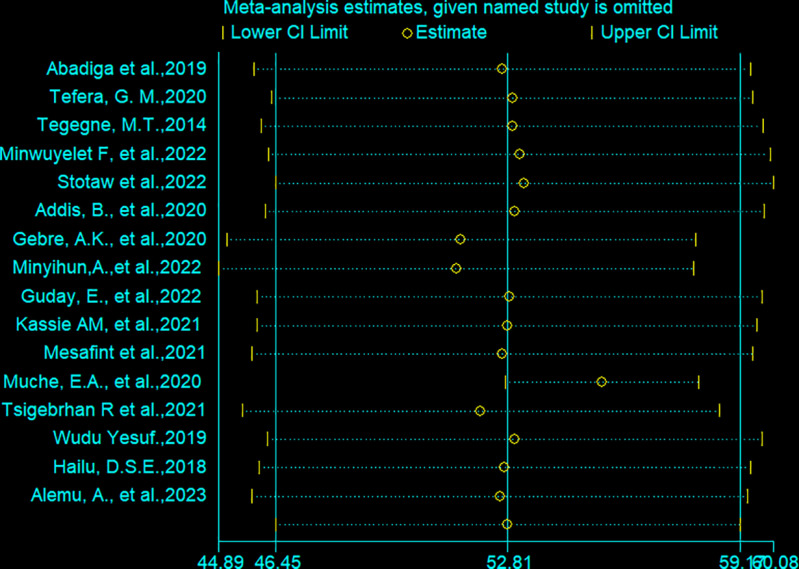
Sensitivity analysis to identify influencing effect of one study on the other.

We have conducted a sensitivity analysis to identify whether there is evidence of the influencing effect of one study on the other. The output of leave-one-out sensitivity analysis through the random-effects model revealed that there was no individual study that influenced the overall pooled estimate of Health-related quality of life of epilepsy patients in Ethiopia HRQOL in this particular review. For every single study, the effect size indicated relates to the overall pooled effect size generated from meta-analysis omitted that particular study (Fig 4).

### Determinants of health-related quality of life of epilepsy patients in Ethiopia

In this systematic review and meta-analysis the variables residency, education, frequency of seizure, seizure control status, perceived stigma, antiepileptic drug adherence, antiepileptic drug side effects, anxiety, depression, social support, and comorbidity were significantly associated with health-related quality of life of epilepsy patients. We reviewed twelve articles [4,19,21,22,24–27,29–32] that had reported statistically significant associations of variables with health-related quality of life. Due to limited articles, we didn’t do a pooled meta-analysis instead put a study with similar variables together and interpreted them as it was in the original article.

Among twelve articles, two [30,31] revealed that rural residency had a significant association with the health-related quality of life of epilepsy patients in Ethiopia. Five studies [19,20,24,27,32] showed that having no formal education had a statistically significant association between health-related quality of life among patients with epilepsy in Ethiopia. Six studies [4,20,25,26,29,32] showed that having seizure frequency had a statistically significant association with health-related quality of life among patients with epilepsy in Ethiopia. Four studies [6,20,22,26] about seizure control status had a statistically significant association between health-related quality of life among patients with epilepsy in Ethiopia. Six studies [4,19–21,25,32] showed that perceived stigma had a statistically significant association with health-related quality of life among patients with epilepsy in Ethiopia. Three studies [4,19,32] on AED adherence and other three studies [4,21,25] on antiepileptic drug side effects showed a statistically significant association between health-related quality of life among patients with epilepsy in Ethiopia. Six studies [4,19–21,24,32] showed anxiety and depression had a statistically significant association between health-related quality of life among patients with epilepsy in Ethiopia. Moreover, seven studies [4,19–21,26,30,33] showed social support had a statistically significant association between health-related quality of life among patients with epilepsy in Ethiopia. Furthermore, five studies [6,19,25,30,33] showed comorbidity had a statistically significant association between health-related quality of life among patients with epilepsy in Ethiopia (S1 Table).

### Evidence Certainty assessment of the included primary studies

In this systematic review and meta-analysis, all the included primary studies were cross-sectional, leading us to a conclusion of low certainty of evidence. The authors further evaluated the certainty using five downgrading domains and three upgrading factors based on the GRADE criteria [35]. The evidence showed some inconsistencies due to the limited number of eligible studies, as reflected by heterogeneity in the estimated outcomes across different regions of Africa. However, no publication or risk of bias was found, as most included studies followed rigorous scientific methods. This was confirmed by Egger’s test and funnel plots, which indicated no publication bias. Additionally, there was no imprecision in outcome measurements, as the studies had sufficient sample sizes and narrow confidence intervals for the pooled effect size. All included studies directly measured the Health-related quality of life of adult epileptic patients without using indirect methods. Potential confounding factors were controlled by addressing all possible influences on the outcome estimate, ensuring reliable results. Overall, the mean HROL among adult epileptic patients in Ethiopia is likely to vary with more studies included. Therefore, further research is needed to provide stronger evidence for informed decision-making (S4 Table).

## Discussion

In this systematic review and meta-analysis, the pooled overall mean score of health-related quality (HRQOL) among epilepsy patients was 52.81 ± 13.24. Moreover, the pooled overall HRQOL mean score using the WHO-QOL-BREF measuring tool was 51.50 ± 11.32**.** This finding was lower than the study conducted in the United Arab Emirates in which the HRQOL overall mean score was 93.3 ± 17.34 [36]. The possible explanation for the variation in overall health-related quality of life mean score might be due to socio-demographic, socio-economic, and socio-cultural variation between patients in Ethiopia and the United Arab Emirates. The finding of the current HRQOL pooled overall mean score using the QOLIE-31 measuring tool was 59.99 ± 12.67 and consistent with multicenter studies conducted in Germany [37], Qassim region [38], and Saudi Arabia [39] in which the mean overall QOLIE-31 score of epilepsy patients was 61.7 ± 18.4, 64.23 ± 17.8, and 61.56 ± 17.52 respectively. It was also consistent with other systematic and meta-analysis studies conducted in China [40] with a pooled overall QOLIE-31 mean score of 65.9 ± 14.25 among adult epileptic patients. However, the pooled overall QOLIE-31 mean score of the current finding was lower than 65.17 ± 19.1 and 77.98 ± 13.32 in the studies conducted in Saudi and Nigeria [41,42] respectively. The possible explanation for the variation in the overall HRQOL means score might be Sociodemographic variation and inclusion criteria difference for instance the study in Saudi excluded those individuals with chronic comorbidity. This perhaps brought about differences in health-related quality of life overall mean score among epileptic patients. However, higher than the finding in Russia [43] with the mean overall QOLIE-31 score of 48.67 ± 10.80. The possible explanation might be because the study in Russia was not a pooled result of primary articles but rather a multicenter individual article among the general population with epilepsy. Furthermore, the current study finding was lower than the finding in America [44] with a pooled overall HRQOL mean score of QOLIE-31 (73.7±) among adult epileptic patients. The variation in the finding might be due to the fact there are socio-demographic, socio-cultural and socio-economical variations between the United States of American patients and patients in Ethiopia. For instance, the patients in the United States of America were more educated and had good antiepileptic drug adherence, on top of this have had a higher pooled overall HRQOL mean score. This could be the possible justification for the variation in overall health-related quality of life mean score. Moreover, the study in the United States of America was only from two registered trials, but the current was the pooled estimate of 16 primary studies. The measuring tool employed for health-related quality of life has an impact on the overall mean score among patients with epilepsy. Regarding domain-based pooled estimates of HRQOL with the QOLIE-31 measuring tool, seizure worry pooled overall QOLIE-31 mean score was (63.93 ± 16.94), consistent with the study conducted in the United States of America [44]. However, the pooled overall quality of life mean score of all other domains such as overall quality of life (62.14 ± 18.26), emotional well-being (63.06 ± 17.68), energy/fatigue (67.90 ± 21.32), cognitive functioning (67.90 ± 20.11), medication effect (68.11 ± 18.76), and social functioning (69.86 ± 23.06) HRQOL mean-scorers were lower than the study conducted in the United States of America. In this regard, the pooled domain-wise HRQOL mean-scorers of the study in the United States of America were overall quality of life (76.0), emotional well-being (79.5), energy/fatigue (70.8), cognitive functioning (73.3), medication effect (73.5), and social functioning (75.2) respectively [44]. The reason for the discrepancy might be due to Sociodemographic and socio-economic variations accounts of the variation in the overall health-related quality of life score and hence the poor health-related quality of life among epileptic patients. Moreover, the current study finding of HRQOL pooled overall mean score of QOLIE-31 tool domains was higher than the study conducted in Portugal with an overall mean score of 54.73 ± 16.30 among adult epilepsy patients [13]. The possible variation might be the time gap and socio-cultural difference between the two populations.

Regarding the factors associated with HRQOL seizure frequency, depression, anxiety, social support, and comorbidity were significant predictors of poor HRQOL among epilepsy patients in Ethiopia. This was supported by the studies conducted in the United Kingdom and United Arab Emirates [36,45**].** Being rural residency was a significant predictor of poor HRQOL overall score as compared to its counterpart. The possible explanation might be those epilepsy patients from rural have had low educational status, low information access about the disease process, and low awareness in general as compared to urban residents with high information access through TV, radio, and even the internet [46]. Furthermore, having no formal education was also a significant predictor of low HRQOL overall mean score among epileptic patients in Ethiopia as compared with formal education. The possible explanation for this might be those with no formal education could probably have a lack of information package as compared to educated ones. This was supported by the study conducted in Jeddah, Saudi Arabia where those patients with low levels of education scored the lowest HRQOL mean score [39]. Those epileptic patients with frequent seizure attacks and uncontrolled seizures were also significant predictors of low HRQOL mean score as compared to no frequent attacks and controlled seizures. The possible explanation might be that individuals with no frequent seizure attacks have had a chance of controlling seizures and their consequences in general, hence they might feel a sense of relaxation as compared to those suffering from frequent seizure attacks since not been controlled yet. This was supported by the studies conducted in the United Arab Emirates and Jeddah, Saudi Arabia in which those individuals with uncontrolled seizures demonstrated the lowest HRQOL mean score as compared to their counterparts [36,37,39].

Besides, having perceived stigma was a significant predictor of low HRQOL means score as compared to its counterpart. This might be because those patients who perceived stigma could not freely share their health problems with the person nearby; they would rather suffer social isolation and low information access as compared to stigma-free individuals. Evidence also supports this, in which individuals with perceived stigma are more likely to have poor health-related quality of life [47,48]. Social support was also a significant predictor of HRQOL of epilepsy patients in Ethiopia in which those with poor or no social support revealed a poor health-related quality of life mean score as compared to those having good social support. The possible explanation might be because those patients with support could not have a sense of isolation and perhaps good antiepileptic drug adherence, and seizure control by far. This was supported by the study/study conducted in Mexico [49]. Anxiety and depression were also significant factors of poor HRQOL of epilepsy patients in Ethiopia in which those with anxiety and depression revealed a poor health-related quality of life mean score as compared to having no anxiety and depression. The reason for this might be those individuals with comorbid anxiety and depression could have poor epilepsy management adherence in general and antiepileptic drug adherence in particular [36,37,42,50,51].

Moreover, comorbidity was a significant predictor for poor health-related quality of life among epilepsy patients in Ethiopia. The possible explanation might be those epileptic patients with other comorbidity conditions could probably be hopeless and non-adherent to their medications and overall therapy, and pose poor health-related quality of life. This was supported by the study conducted in Japan [52]. The findings of this systematic review and meta-analysis study have a significant contribution for those concerned about epilepsy and its determinants to take remedies. Emphasized has to be given to this cohort of population to increase awareness about the disease process to have a great resilience, and to decrease comorbid conditions.

### Limitations and strengths of the study

This systematic review and meta-analysis has limitations firstly it included only articles from Ethiopia, and published only in the English language. The study also did not present the pooled effect size of factors associated with health-related quality of life due to limited data. The variation among the included studies in the meta-analysis could be seen as both strength and weakness. However, in our case we authors thought that the variation among included primary articles in terms of sample size, study setting, and estimates of the outcome of interest led to an increase in generalizability and gave us deeper insights. As far as the authors handled it appropriately through subgroup analysis, sensitivity analysis, trim and fill analysis, and meta-regression analysis. On the other hand, it could be a weakness given that the variation leads to excessive heterogeneity or poses a challenge to the interpretation of pooled estimates generated. Besides, what matters is the way the authors properly assess and give due consideration to this variability in the statistical methods employed such as random-effects models and in the interpretation of findings. Overall, the studies we included in the current systematic review and meta-analysis were not greatly differing in terms of techniques or populations hence it was not difficult to draw a conclusion that was again a strength, not a weakness.

### Implications of the study

This systematic review and meta-analysis study has crucial benefits for those who are involved in epileptic care directly and indirectly. For instance, it helps the health care providers, policymakers, and program planners by providing current and up-to-date information regarding the health-related quality of life of patients with epilepsy. It also helps to set off possible strategies to improve the quality of life of patients as well as families by providing insight information how to minimize other comorbid psychiatric disorders. Hence, minimizes disease burden and a cost demanded from the patient, family, and the country at large.

## Conclusion

The lowest overall pooled mean score of HRQOL was the QOLIE-10 measuring tool epileptic patients’ mean score as compared to other health-related quality-of-life measuring tools such as WHOQOL-BREF, and QOLIE-31. Moreover, the least overall pooled mean score of HRQOL was the environmental domain followed by the emotional domain as compared to all other domains in each measuring tool. Being rural residents, not educated, not adhering to antiepileptic drugs, having AE drug side effects, having perceived stigma, anxiety, depression, and comorbidity were significant predictors of low health-related quality of life among epilepsy patients in Ethiopia. Therefore, there have to be possible strategies set off to improve the quality of life of patients as well as families by providing insight information on how to minimize other comorbid psychiatric disorders.

## Supporting information

S1 File This is the PRISMA CHECKLIST. (DOCX)

S2 File This is the Data Availability Statement. (XLSX)

S3 File This is all data extracted. (ZIP)

S4 File This is the Missed data handling mechanism. (DOCX)

S1 Table This is the determinants of health-related quality of life of epilepsy patients in Ethiopia. (DOCX)

S2 Table This showed all studies identified in the literature search, including those that were excluded from the analyses. (DOCX)

S3 Table This is a table of all data extracted from the primary research sources for the systematic review and/or meta-analysis. (DOCX)

S4 Table This is the Grade Score of included studies in the final systematic Review and Meta-analysis. (DOCX)

S1 Fig This is a baujat plot to identify outliers among the included studies. (TIFF)

S2 Fig This is a bubbles plot to show the sources of heterogeneity among the included studies. (TIFF)

## Abbreviations

HADS: Hospital Anxiety and Depression Scale
HRQOL: Health-related Quality of Life
JPSS-3: three-item Jacoby perceived stigma scale
KSSE-3: The Kilif Stigma Scale of Epilepsy
LAEP: Liverpool Adverse Events Profile
MARS-5: Medication Adherence Reporting Scale-5
Oslo: Social support Scale
QOLIE-10: Quality of Life in Epilepsy Inventory-10
QOLIE-31: Quality of Life in Epilepsy Inventory-31
rESS-3: the three item revised version of the Epilepsy Stigma Scale
WHOQOL-BREF: World Health Organization Quality of Life—Brief
