# Supplementary material for: Health-related quality of life and its predictors among epilepsy patients in Ethiopia: Systematic review and meta-analysis
Source: PLoS One. 2025 Jun 3;20(6):e0324363. doi: 10.1371/journal.pone.0324363 (PMC12132937; doi:10.1371/journal.pone.0324363)
Supplement: S1 File — (DOCX) [file pone.0324363.s001.docx]

| **Section and Topic** | **Item #** | | **Checklist item** | **Location where item is reported** |
| --- | --- | --- | --- | --- |
| **TITLE** | | | |  |
| Title | 1 | | **Health-related quality of life and its predictors among epilepsy patients in Ethiopia: Systematic Review and Meta-analysis** | 1 |
| **ABSTRACT** | | | |  |
| Abstract | 2 | | **Abstract**  **Introduction:** Epilepsy is a common non-communicable neurological disorder associated with recurrent seeding of cerebral neurons or brain cells and episodes of unprovoked seizures with or without loss of consciousness. Although there are studies on the health-related quality of life of epilepsy patients in Ethiopia, there are remarkable variations in the estimates of health-related quality of life associated with measurement differences and reporting. Therefore, this systematic review and meta-analysis aimed to generate aggregated and rigorous data on the health-related quality of life of epilepsy patients in Ethiopia.  **Objectives:** To determine the pooled effect size of health-related quality of life of epilepsy patients in Ethiopia.  **Method**: Original articles about the health-related quality of life among epilepsy patients in Ethiopia were searched through known and international databases (PubMed, Scopus, Web of Science) and search engines (google and Google Scholar). Data were extracted using a standard data extraction checklist that was developed according to Joanna Briggs Institute (JBI). The I^2^ statistics were used to identify heterogeneity across studies. Funnel plot asymmetry and Egger's tests were used to check for publication bias. A Random effect model was used to estimate the pooled prevalence of depression among medical students in Africa. Statistical analysis was conducted using STATA version 11 software.  **Result:** A total of 16 cross-sectional observational studies with a 5294 sample population took part which provided information about health-related quality of life among epilepsy patients in Ethiopia. The pooled overall means score of health-related quality of life among epilepsy patients in Ethiopia was 57.97±SD [95%CI (51.22, 64.71)], I2 =96.3%, p-value <0.001. Whereas, the overall pooled mean score of health-related quality of life based on the measuring tools WHO-QOL-BREF, QOLIE-31, and QOLIE-10 was 56.73±SD (54.56, 58.95)**,** 64.15±SD (56.34, 71.96), and 44.73±SD (41.2, 93.58) respectively.  **Conclusion:** The least overall pooled mean score of HRQOL was the QOLIE-10 measuring tool epileptic patients' mean score as compared to other health-related quality-of-life measuring tools such as WHOQOL-BREF, and QOLIE-31. Moreover, the least overall pooled mean score of HRQOL was the environmental domain followed by the emotional domain, as compared to all other domains in each measuring tool of health-related quality of life.  **Keywords:** Health-related quality of life; Quality of Life, Epilepsy, Adults, Mental health Ethiopia | 2 |
| **INTRODUCTION** | | | |  |
| Rationale | 3 | | **Introduction**  Epilepsy is defined as a common non-communicable neurological disorder associated with recurrent seeding of cerebral neurons or brain cells and episodes of unprovoked seizures with or without loss of consciousness [1]. However, people who have had only seizure related to other well-defined febrile conditions is not considered as epilepsy [2]. Health-related quality of life (HRQOL) is a complex and multidimensional conception which encompasses physical health, psychological state, social, and environmental maladjustments[3]. Studies conducted on health-related quality of life of epilepsy patients become increasingly common in low and middle-income countries, particularly in Ethiopia. However, there are remarkable variations in the estimates of health-related quality of life associated with measurement differences and reporting [4-6]. In Sub-Saharan Africa, a prevalence of 9.39 per 100 epileptic cases was found [7]. Furthermore, the global prevalence of epilepsy is reported that there are over 50 million individuals suffering from epilepsy and of these more than 125,000 die each year[8]. According to the Global Burden of Epilepsy Report, nearly 13 million adjusted life years disabilities occur due to epilepsy annually [9]. Evidence in the US revealed that the prevalence of idiopathic epilepsy declined from 76.9% to 75.1 between 1999 and 2017[10]. Epilepsy has a remarkable impact on patient's well-being, social capital and health-related quality of life due to various contributing factors such as medication adherence, socio-economic cost, decreased employment probability, and comparatively low income [11]. Moreover, individuals with epilepsy are prone to social isolation or stigma related to decreased self-esteem, comorbid depression, anxiety, and suicidal attempts as a result of predicting recurrent seizure episodes which decline the health-related quality of life of patients[12]. Epilepsy patients have physical, psychological, cognitive, social, and environmental maladjustments which result in a significant loss of health-related quality of life [13]. There are disparities in the health-related quality of life among studies conducted in Ethiopia. Therefore, this systematic review and meta-analysis tried to minimize the variation by generating the pooled estimates of individual studies. | 4 |
| Objectives | 4 | | To determine the pooled effect size of health-related quality of life of epilepsy patients in Ethiopia. | 2 |
| **METHODS** | | | |  |
| Eligibility criteria | 5 | | In this review the primary articles were eligible if and only if (1) they reported epilepsy patients (2) aged≥ 18 years old (3) those articles reported the overall mean score of health-related quality of life (4) observational studies (both analytical and descriptive cross-sectional) (5) articles published in English and only from Ethiopia were included, and (6) published from 2014 to 2023 were included in this particular systematic review and Meta-analysis. Nonetheless, after a thorough examination of the titles and abstracts using eligibility criteria, irrelevant studies such as conference reports, qualitative papers, and published in languages other than English were excluded. | 5 |
| 5Information sources | 6 | | The known and international databases (PubMed, Scopus, Web of Science, and Cochran library) and searching engines (goggle and Google scholar) were used to locate research articles on health-related quality of life of epilepsy patients in Ethiopia. | 5 |
| Search strategy | 7 | | The string for searching was developed using “AND” and “OR” Boolean operators with the keywords extracted from the Medical Subject Headings (MeSH) database. The search strategy was based on the research question of this review and utilized the **CoCoPop (Co=**Condition**, Co=**Context**, Pop=**Population**) model.**  The article locating strategy was through "health-related quality of life" **OR** HRQOL **OR** "epilepsy patients HRQOL" **OR** "quality of life" **OR** "epilepsy patient* QOL" **AND** "epilepsy patient*" **OR** "adult epilepsy patients" **OR** "Seizure disorder*" **OR** *Seizure **AND** *Ethiopia **OR** Ethiopia. This search strategy primarily aimed to trace all reviewed (published) and unpublished primary studies**.** The list of all retrieved primary articles and systematic review and meta-analyses references were also screened or cross referenced to get extra studies. The sources of information range from electronic databases to direct contact with the principal investigator if mandatory. The first search through Pub Med, Cochran Library, Scopus, Web of Science, Google, and Google Scholar was done in November 2023. The final search for updating was conducted from **November 20 / 2023 to December 20/ 12/2023**. The publication date was used as a filter mechanism in which articles published from January **2014 to December 2023** were included in the current systematic Review and Meta-analysis study to generate the most recent evidence for the scientific community. | 5 |
| Selection process | 8 | | After comprehensive searching, all located citations were selected and exported to Endnote citation manager software version X7. Following this, irrelevant and duplicated articles were removed. Then three independent researchers (HKA, MCA and AWA) screened each particular article for its title, abstract, and full text by far and cross-checked it against the inclusion criteria. The other research team (CKM, MCA, and AWA) checked the screened articles with full text for details by already defined criteria to take it to the final review process. Any sort of disagreement between the research team while including and excluding articles on predefined criteria of this particular review was resolved by a thorough discussion of the team. The exclusion of the articles was presented with countable reasons which could be consistent with the pre-defined criteria. The result of searching further screening and inclusion process of articles in this review was done in agreement with the PRISMA guidelines for Systematic Review and Meta-analysis 2020. | 6 |
| Data collection process | 9 | | Data were independently extracted by five authors using a standardized data extraction format that was developed according to the 2014 Joanna Briggs Institute Reviewers’ Manual [17]. The tool includes Authors, Region, study year, study design, sample size, the mean score of health-related quality of life of epilepsy patients, a tool used to measure the outcome, response rate, and risk of bias assessment score included in the extraction. The data were extracted by two independent reviewers and any inconsistent data was cross-checked (Additional File 2). The disagreement between the reviewers was solved by a thorough discussion. | 7 |
| Data items | 10a | | Articles with clear outcomes about health-related quality of life, and published from 2013 to 2023 were included in this particular systematic review and Meta-analysis. | 7 |
|  | 10b | | Articles with clear outcomes about health-related quality of life and low risk of bias | 7 |
| Study risk of bias assessment | 11 | | There are a total of 31 articles assessed for methodological quality using 9 point score tool developed by JBI for observational prevalence studies. The outcome of the quality appraisal ranged from moderate to high methodological quality in which eleven studies[22-32] score 9 points, eleven studies[15, 33-42] scored 8 points, four studies[14, 43-45] scored 7 points and the remaining five studies[46-50] scored 6 points.( **Additional File 1**). | 8 |
| Effect measures | 12 | | The odds ratio (OR), LogM, and standard error of logM or (SelogM) were used presentation of results. | 8 |
| Synthesis methods | 13a | | The qualitative and quantitative synthesis was employed. | 8 |
|  | 13b | | The pooled effect-size with random model of analysis was employed. | 8 |
|  | 13c | | We have used the PRISMA flow chart, forest plot, and funnel plot to present and visually displayed the data. | 8 |
|  | 13d | | The standard chi-square and I-square statical tests were used. The variation between different studies characteristics such as the country where the primary article conducted and outcome ascertainment tool were investigated through subgroup analyses. This subgroup analyses could demonstrate the sources of heterogeneity and let the researcher for another remedy such as the use of meta-regression to treat this heterogeneity. The influence of individual articles on the overall pooled effect size estimate or health-related quality of life of epilepsy patients was assessed by using the sensitivity analysis. The forest plot with 95% CI was used to present the overall pooled mean score as well as the subgroup pooled health-related quality of life of epilepsy patients in Ethiopia. | 12 |
|  | 13e | | A random -effects model was used for analysis. | 13 |
|  | 13f | | We have conducted a sensitivity analysis to identify whether there is evidence of the influencing effect of one study on the other. The output of leave-one-out sensitivity analysis through the random-effects model revealed that there was no individual study that influenced the overall pooled estimate of Health-related quality of life of epilepsy patients in Ethiopia HRQOL in this particular review. For every single study, the effect size indicated relates to the overall pooled effect size generated from meta-analysis omitted that particular study (Fig.4). | 15 |
| Reporting bias assessment | 14 | | We have used the Begg’s and/or Egger’s test to detect publication bias. | 15 |
| Certainty assessment | 15 | | The forest plot with 95% CI was used to present the overall pooled mean score of health-related quality of life as well as the subgroup pooled mean score epilepsy patients in Ethiopia. | 11 |
| **RESULTS** | | | |  |
| Study selection | 16a | | In this systematic review and meta-analysis study a total of (1654) articles related to the quality of life of epilepsy patients in Ethiopia were identified using electronic databases and search engine websites. Among overall articles, 1094 were removed for being irrelevance and duplicated and the other pretty sizable articles were removed for not being ineligible (study design and Title difference) by automation tools and other reasons (402 vs. 77) respectively. The remaining 81 articles were eligible for screening. Of these screened 49 papers were excluded due to the country of study or not being conducted in Ethiopia and target population difference (those articles conducted among children). With further screening, 32 articles were sought for retrieval and 8 were not retrieved for one and the other reason. Moreover, 24 research articles were assessed for eligibility to be included in the review process, but with the outcome of interest and measurement tool ambiguity a total of 8 articles were excluded. Finally, 16 original research articles were incorporated with the systematic review and meta-analysis (**Fig. 1**). | 8 |
|  | 16b | | After a thorough examination of the titles and abstract using eligibility criteria, irrelevant studies were excluded. Then those articles considered relevant were reviewed; whereas those without clear reporting of the outcome of interest were excluded. | 6 |
| Study characteristics | 17 | | In this systematic review and meta-analysis, 5294, participants were included with a response rate of 100%. The studies included in this review were observational cross-sectional studies published from 2014 to 2023. The smallest sample size was 78 from the study conducted in the Amhara region, Ethiopia [22] followed by 121 from the Oromia, region of Ethiopia [30]. Whereas, the largest sample was 462 from Amhara region, Ethiopia [18] followed by 439 the study from Addis Ababa, Ethiopia[4]. Moreover, eight studies[4, 18, 20, 23-25, 29, 31] used WHOQOL-BREF measuring, six studies[19-21, 23, 26, 27] used QOLIE-31, and the rest two studies[28, 33] used QOLIE-10 measuring tool to measure health-related quality of life of epilepsy patients. Besides, the statistical models employed were linear regression and logistic regression in which four studies [23, 25, 29, 31] used logistic regression and the remaining twelve studies used linear regression (Table 2). | 9 |
| Risk of bias in studies | 18 | | There are a total of 31 articles assessed for methodological quality using 9 point score tool developed by JBI for observational prevalence studies. For the nutshell, all articles had had high quality and included in the final analysis process. |  |
| Results of individual studies | 19 | | \| Author/reference \| Publication year \| Region \| Study design \| Sample size \| Measuring tool \| Statistical model \| \| --- \| --- \| --- \| --- \| --- \| --- \| --- \| \| Abadiga et al.[24] \| 2019 \| Oromia, Ethiopia \| Crossectional \| 392 \| WHOQOL-BREF \| Linear regression \| \| Tefera, G. M.[30] \| 2020 \| Oromia, Ethiopia \| Crossectional \| 121 \| WHOQOL-BREF \| Linear regression \| \| Tegegne, M.T.[31] \| 2014 \| Oromia, Ethiopia \| Crossectional \| 415 \| WHOQOL-BREF \| Logistic regression \| \| Minwuyelet F, et al.[23] \| 2022 \| Amhara, Ethiopia \| Crossectional \| 402 \| WHOQOL-BREF \| Logistic regression \| \| Stotaw et al.[29] \| 2022 \| Amhara, Ethiopia \| Crossectional \| 384 \| WHOQOL-BREF \| Logistic regression \| \| Addis, B., et al.[20] \| 2021 \| Amhara, Ethiopia \| Crossectional \| 370 \| QOLIE-31 \| Linear regression \| \| Gebre, A.K., et al.[26] \| 2018 \| Tigray, Ethiopia \| Crossectional \| 175 \| QOLIE-31 \| Linear regression \| \| Minyihun,A.,et al. [22]. \| 2022 \| Amhara, Ethiopia \| Crossectional \| 78 \| QOLIE-31 \| Linear regression \| \| Guday, E., et al. [18] \| 2022 \| Amhara, Ethiopia \| Crossectional \| 462 \| WHOQOL-BREF \| Linear regression \| \| Kassie AM, et al. [21] \| 2021 \| Amhara, Ethiopia \| Crossectional \| 395 \| QOLIE-31 \| Linear regression \| \| Mesafint et al.[4] \| 2020 \| Addis Ababa, Ethiopia \| Crossectional \| 439 \| WHOQOL-BREF \| Linear regression \| \| Muche, E.A., et al. [28] \| 2020 \| Amhara, Ethiopia \| Crossectional \| 354 \| QOLIE-10 \| Linear regression \| \| Tsigebrhan, R., et al.[33] \| 2021 \| Southern, Ethiopia \| Crossectional \| 237 \| QOLIE-10 \| Linear regression \| \| Wudu Yesuf.[19] \| 2019 \| Oromia, Ethiopia \| Crossectional \| 340 \| QOLIE-31 \| Linear regression \| \| Hailu, D.S.E.[27] \| 2018 \| Oromia, Ethiopia \| Crossectional \| 304 \| QOLIE-31 \| Linear regression \| \| Alemu, A., et al[25]. \| 2023 \| Southern, Ethiopia \| Crossectional \| 423 \| WHOQOL-BREF \| Logistic regression \| |  |
| Results of syntheses | 20a | | We have assessed the risk of bias using Begg’s and/or Egger’s test. | 15 |
|  | 20b | | A total of 16 primary articles were appraised and retrieved to pool the overall mean score of health-related quality of life among epilepsy patients in Ethiopia. The mean score of health-related quality of life overall score of individual studies ranged from 19.85±6.91 to 79.14±25.46 in the Amhara Region, Ethiopia respectively. The pooled overall means score of health-related quality of life among epilepsy patients in Ethiopia was 57.97±SD [95%CI (51.22, 64.71)], I2 =96.3%, p-value <0.001. The effect size of the overall pooled mean score of health-related quality of life among epilepsy patients in Ethiopia was presented using a forest plot (Fig.2). | 11 |
|  | 20c | | In this systematic review and meta-analysis, the analysis output using random-effects model showed a high variability across the primary articles included in the study(I^2^= 100%, P<0.001). This variability is inevitable in meta-analysis studies resulting from quality difference of the included studies, methodological differences, sample size, inclusion and exclusion, and the difference in measuring tool to ascertain the outcome of interest. Therefore, we have conducted the meta-regression analysis by using publication year, sample size, and standard error as a covariates to figure out the potential source of heterogeneity among included studies. | 14 |
|  | 20d | | We have conducted a sensitivity analysis to identify whether there is an evidence of influencing effect of one study on the other. The output of leave-one-out sensitivity analysis through random-effects model revealed that there was no any individual study that influenced the overall pooled mean score estimate of health-related quality of life of epilepsy patients in this particular review. For each single study, the effect size indicated relates with the overall pooled effect size generated from meta-analysis omitted that particular study **(Fig. 5**). | 15 |
| Reporting biases | 21 | | In this systematic review and meta-analysis, the analysis output using a random-effects model showed high variability across the primary articles included in the study (I2= 96.3%, P<0.001). This variability is inevitable in meta-analysis studies resulting from quality differences of the included studies, methodological differences, sample size, inclusion and exclusion, and the difference in measuring tools to ascertain the outcome of interest. Therefore, we have conducted the meta-regression analysis by using publication year, sample size, and standard error as covariates to figure out the potential source of heterogeneity among included studies. In this regard, the meta-regression analysis revealed that no significant correlation was found between the outcome of interest (HRQOL) and the included covariates by far (p =0.997, for publication year and P= 0.932 for sample size). Hence, there was no statistically significant association and possible existence of variability as shown (Table 4). This again implies that the source of high variability (heterogeneity) could be due to chance or the other variables not investigated in this particular review. | 14 |
| Certainty of evidence | 22 | | The mean score of health-related quality of life overall score of individual studies ranged from 19.85±6.91 to 79.14±25.46 in the Amhara Region, Ethiopia respectively. The pooled overall means score of health-related quality of life among epilepsy patients in Ethiopia was 57.97±SD [95%CI (51.22, 64.71)], **I^2^ =96.3%, p-value <0.001.** The effect size of the overall pooled mean score of health-related quality of life among epilepsy patients in Ethiopia was presented using a forest plot (**Fig.2).** The pooled mean score of health-related quality of life-based on the measuring tools WHO-QOL-BREF, QOLIE-31, and QOLIE-10 by domain was done. Hence, WHOQOL-BERF has four domains of health such as physical domain of health, psychological domain of health, Social domain of health, and environmental domain of health. The domain based pooled mean-score was physical 52.67±SD [95%CI(40.70, 64.64)], I^2^ =96.3%, p-value <0.001**,** psychological 50.50±SD [95%CI(39.26, 65.73)], I^2^ =100%, p-value <0.001, emotional 49.97±SD [95%CI(32.34, 67.61), I^2^ =100%, p-value <0.001**,** and environmental 48.09±SD [95%CI(38.31, 57.87)], I^2^ =100%, p-value <0.001 respectively. The lowest and highest mean scores were in the environmental and physical health domains respectively. Regarding, the QOLIE-31 measuring tool, it has seven domains with the highest and lowest mean scores of health-related quality of life 69.86±SD [95%CI (62.99, 76.73)] in the domain of social functioning and 62.14 ±SD [95%CI (57.11, 67.17)] in the overall quality of domain respectively (Table 3). | 11-15 |
| **DISCUSSION** | | | |  |
| Discussion | 23a | | Discussion  In this systematic review and meta-analysis, the pooled overall mean score of health-related quality (HRQOL) among epilepsy patients was 57.97±SD [95%CI (51.22, 64.71)], I2 =96.3%, p-value <0.001. Moreover, the pooled overall HRQOL mean score using the WHO-QOL-BREF measuring tool was 56.73±SD [95%CI (54.50, 58.95)], I2 =36.2%, p-value <0.001. This finding was lower than the study conducted in the United Arab Emirates in which the HRQOL overall mean score was 93.3 ±SD [35]. The possible explanation for the variation in overall health-related quality of life mean score might be due to socio-demographic, socio-economic, and socio-cultural variation between patients in Ethiopia and the United Arab Emirates. The finding of the current HRQOL pooled overall mean score using QOLIE-31 measuring tool was 64.15±SD (56.34, 71.96) and consistent with multicenter studies conducted in Germany [36], Qassim region[37], and Saudi Arabia [38] in which the mean overall QOLIE-31 score of epilepsy patients was 61.7±SD, 64.23±SD, and 61.56±SD respectively. It was also consistent with other systematic and meta-analysis studies conducted in China [39] with pooled overall QOLIE-31 mean score of 65.9 ±SD among adult epileptic patients. However, the pooled overall QOLIE-31 mean score of the current finding was lower than 72.68±SD and 77.98±SD in the studies conducted in Saudi and Nigeria [40, 41] respectively. The possible explanation for the variation in the overall HRQOL means score might be Sociodemographic variation and inclusion criteria difference for instance the study in Saudi excluded those individuals with chronic comorbidity. This perhaps brought about differences in health-related quality of life overall mean score among epileptic patients. However, higher than the finding in Russia [42] with the mean overall QOLIE-31 score of 48.67±SD. The possible explanation might be because the study in Russia was not a pooled result of primary articles but rather a multicenter individual article among the general population with epilepsy. Furthermore, the current study finding was lower than the finding in America [43] with a pooled overall HRQOL mean-score of QOLIE-31 (73.7±SD) among adult epileptic patients. The variation in the finding might be due to the fact there are socio-demographic, socio-cultural and socio-economical variations between the United States of American patients and patients in Ethiopia. For instance, the patients in the United States of America were more educated and had good antiepileptic drug adherence, on top of this have had higher pooled overall HRQOL mean score. This could be the possible justification for the variation in overall health-related quality of life mean-score. Moreover, the study in the United States of America was only from two registered trials, but the current was the pooled estimate of 16 primary studies. The measuring tool employed for health related quality of life has an impact on the overall mean-score among patients with epilepsy. Regarding domain based pooled estimates of HRQOL with QOLIE-31 measuring tool, seizure worry pooled overall QOLIE-31 mean score was (63.93±SD), consistent with the study conducted in the United States of America [43]. However, the pooled overall quality of life mean score of all other domains such as overall quality of life (62.14 ±SD), emotional well-being (63.06±SD), energy/fatigue (67.90±SD), cognitive functioning (67.90±SD), medication effect (68.11±SD), and social functioning (69.86±SD) HRQOL mean-scorers were lower than the study conducted in the United States of America. In this regard, the pooled domain wise HRQOL mean-scorers of the study in United States of America were overall quality of life (76.0 ±SD), emotional well-being (79.5±SD), energy/fatigue (70.8±SD), cognitive functioning (73.3±SD), medication effect (73.5±SD), and social functioning (75.2±SD) respectively [43]. The reason for the discrepancy might be due to Sociodemographic and socio-economic variations accounts of in the variation in the overall health-related quality of life score and hence the poor health-related quality of life among epileptic patients. Moreover, the current study finding of HRQOL pooled overall mean score of QOLIE-31 tool domains were higher than the study conducted in Portugal with the overall mean score of 54.73±SD among adult epilepsy patients [13]. The possible variation might be the time gap and socio-cultural difference between the two populations. The measuring tool employed for health related quality of life has an impact on the overall mean-score among patients with epilepsy. (SF-36 Vs QOLIE-31) females associated with [40].  Regarding the factors associated with HRQOL seizure frequency, depression, anxiety, social support, and comorbidity were significant predicators of poor HRQOL among epilepsy patients in Ethiopia. This was supported by the studies conducted in United Kingdom and United Arab Emirates [35, 44]. Being rural residency was a significant predictor of poor HRQOL overall score as compared to its counterpart. The possible explanation might be those epilepsy patients from rural have had low educational status, low information access about the disease process, and low awareness in general as compared to urban residents with high information access through TV, radio, and even internet [45]. Furthermore, having no formal education was also a significant predictor of low HRQOL overall mean score among epileptic patients in Ethiopia as compared with formal education. The possible explanation for this might be those with no formal education could probably have lack of information package as compared to educated ones. This was supported by the study conducted in Jeddah, Saudi Arabia where those patients with low level of education score the lowest HRQOL mean score[38]. Those epileptic patients with frequent seizure attack and uncontrolled seizure were also significant predictors of low HRQOL mean score as compared to no frequent attack and controlled seizure. The possible explanation might be due to the fact that individuals with no frequent seizure attack have had a chance of controlling seizure and its consequence in general, hence the might feel a sense of relax as compared to those suffering with frequent seizure attack since not controlled yet. This was supported by the studies conducted in the United Arab Emirates and Jeddah, Saudi Arabia in which those individuals with uncontrolled seizure demonstrated the lowest HRQOL mean score as compared to its counterpart [35, 36, 38].  Besides, having perceived stigma was a significant predictor of low HRQOL means score as compared to its counterpart. This might be due to the fact that those patients perceived stigma could not feely share their health problems with the person nearby; they would rather suffer social isolation and low information access as compared to stigma free individuals. Evidence also supports this, in which individuals with perceived stigma are more likely to have poor health related quality of life [46, 47]. Social support was also significant predictor of HRQOL of epilepsy patients in Ethiopia in which those with poor or no social support revealed a poor health-related quality of life mean score as compared to having good social support. The possible explanation might be due to the fact that those patients with support could not have sense of isolation and perhaps good antiepileptic drug adherence, and seizure control by far. This was supported by the study/study conducted in Mexico [48]. Anxiety and depression were also significant factors of poor HRQOL of epilepsy patients in Ethiopia in which those with anxiety and depression revealed a poor health-related quality of life mean score as compared to having no anxiety and depression. The reason for this might be those individuals with comorbid anxiety and depression could have poor epilepsy management adherence in general and antiepileptic drug adherence in particular [35, 36, 41, 49, 50].  Moreover, comorbidity was a significant predictor for poor health-related quality of life among epilepsy patients in Ethiopia. The possible explanation might be due to the fact that those epileptic patients with other comorbidity conditions could probably be hopeless and non-adhere to their medications and overall therapy, and poses poor health-related quality of life. This was supported by the study conducted in Japan [51]. The findings of this systematic review and meta-analysis study have significant contribution for those concerned about epilepsy and its determinants to take remedies. Emphasized has to be given to this cohort of population to increase awareness about the disease process to have a great resilience, to decrease comorbid conditions. This systematic review and meta-analysis has limitations firstly it included only articles from Ethiopia, and published only in English language. The study also did not present the pooled effect size of factors associated with health-related quality of life due to limited data. | 17-20 |
|  | 23b | | However this review has its limitations such as it included only articles published in English were included in this systematic review and meta-analysis**.** The primary studies were all observational cross-sectional studies | 20 |
|  | 23c | | In addition, the meta-analysis didn’t include pooled effects of the factors attributed for poor health-related quality of life of epilepsy patients’ in Ethiopia. It didn’t address all the global evidence published elsewhere with any language. | 20 |
|  | 23d | | This systematic review and meat-analysis study has crucial benefits for those who involved in epileptic care directly and indirectly. For instance, it helps the health care providers, policymakers, and program planners through providing current and up-to-date information regarding health-related quality of life of patients’ with epilepsy. It also helps to set off possible strategies to improve the quality of life of patients as well as families by providing insight information how to minimize other comorbid psychiatric disorders. Hence, minimize disease burden and a cost demanded from the patient, family, and the country at large. | 20 |
| **OTHER INFORMATION** | | | |  |
| Registration and protocol | 24a | This systematic review and meta-analysis title and its protocol were registered in the PROSPERO online database (with registration number CRD420234880041). | | 5 |
|  | 24b | This particular review protocol can be accessed via online databases. | |  |
|  | 24c | The amendments for this review might be done /might not be done. | |  |
| Support | 25 | All the Authors did not receive any fund for to accomplish this review study so far. | |  |
| Competing interests | 26 | The authors have declared that there are no competing interests. | |  |
| Availability of data, code and other materials | 27 | The data extracted for this review was analysed and reported in the main document. | |  |

*From:*  Page MJ, McKenzie JE, Bossuyt PM, Boutron I, Hoffmann TC, Mulrow CD, et al. The PRISMA 2020 statement: an updated guideline for reporting systematic reviews. BMJ 2021;372:n71. doi: 10.1136/bmj.n71

For more information, visit: <http://www.prisma-statement.org/>
