# Supplementary material for: Health-related quality of life and its predictors among epilepsy patients in Ethiopia: Systematic review and meta-analysis
Source: PLoS One. 2025 Jun 3;20(6):e0324363. doi: 10.1371/journal.pone.0324363 (PMC12132937; doi:10.1371/journal.pone.0324363)
Supplement: S4 File — (DOCX) [file pone.0324363.s010.docx]

**Missed data handling mechanism**

In this systematic review and meta-analysis, we made every effort to minimize missing data to ensure the results are rigorous and scientifically reliable. As it understandable, handling missing data is commanded for ensuring reliability and validity of findings. As missing data might occur at the study level including missed outcomes, sample size, or effect sizes or at the participant level within individual studies, data handling has to be crucial. Furthermore, as it known that missing data result in decrease precision and possibly biased of the effect estimates of single studies. In fact, there was no missing among the included primary articles in our systematic Review and Meta-analysis study because we included them all and pooled estimate was generated. This is since the authors have had an extensive searching of articles in order not to miss data by making our searching and pre-define criteria for including and excluding the primary articles clear and precise. The Authors have used a standardized data extraction form for each single study included and also conducted a robust inspection of our data before formal analysis took over. Moreover, the authors have conducted sensitivity and trim and fill analysis to detect and adjust potential publication bias.
