# Supplementary material for: Health-related quality of life and its predictors among epilepsy patients in Ethiopia: Systematic review and meta-analysis
Source: PLoS One. 2025 Jun 3;20(6):e0324363. doi: 10.1371/journal.pone.0324363 (PMC12132937; doi:10.1371/journal.pone.0324363)
Supplement: S2 Table — (DOCX) [file pone.0324363.s005.docx]

**S1 Table**: Predictors of health-related quality of life of epilepsy patients in Ethiopia

| **Determinants** | Exposure variables measurement | Author(reference) | Sample size | Scale /measurement | Overall mean score of HRQOL | Effect size | Comments |
| --- | --- | --- | --- | --- | --- | --- | --- |
| Residence(urban) | **Questionnaire** | Tefera, G. M.[19] | 121 | WHOQOL-BREF | 56.42±10.96 | B=-3.22  P=0.03 | Being rural has a three times decrease in HRQOL mean score as compare to urban |
|  |  | Stotaw et al.[22] | 384 | WHOQOL-BREF | 51.98±10.08 | OR=7.07  P= 0.001 | Being rural has seven times more likely to have poor HRQOL with decrease mean score as compare to urban |
| No formal education | **Questionnaire** | Tefera, G. M.[19] | 121 | WHOQOL-BREF | 56.42±10.96 | B=-8.72  P=0.001 | Having no formal education showed nearly a 9 time decreased in HRQOL mean score |
|  |  | Minwuyelet F, et al.[21] | 402 | WHOQOL-BREF | 53.47±18.42 | OR=2.51(1.19, 5.28)* | People with no formal education were 2.51 times more likely to have to have poor HRQOL with decrease mean score as compare to formal education. |
|  |  | Gebre, A.K., et al.[24] | 175 | QOLIE-31 | 77.98±20.78 | R=0.299  P=0.001 | Education had significant correlation with HRQOL mean-score. |
|  |  | Tegegne, M.T.[20] | 415 | WHOQOL-BREF | 56.36±13.37 | OR=4.69(1.38, 15.87) | People with no formal education were nearly five times more likely to have to have poor HRQOL with decrease mean score as compare to formal education. |
|  |  | Wudu Yesuf.[30] | 340 | QOLIE-31 | 55.6 ±20.9 | B=-4.09  (-6.87, -1.31) | People with no formal education had a four times decrease in HRQOL mean score as compare to formally educated ones. |
| Frequency of seizure | Yes/No | Mesafint et al.[4] | 439 | WHOQOL-BREF | 61±11.6 | B=-3.16  (-4.22, -2.04) | People with frequent seizure had a three times decrease in HRQOL mean score as compare to no frequent seizure. |
|  |  | Tegegne, M.T.[20] | 415 | WHOQOL-BREF | 56.36±13.37 | OR=2.25(1.19, 4.26) | People with frequent seizure were 2.25 three times more likely to have poor HRQOL mean score as compare to no frequent seizure |
|  |  | Alemu, A., et al[32]. | 423 | WHOQOL-BREF | 56.17±10.01 | OR=5.2  P=0.001 | People with frequent seizure were 2.25 three times more likely to have poor HRQOL mean score as compare to no frequent seizure. |
|  |  | Abadiga et al.[18] | 392 | WHOQOL-BREF | 60.47±23.07 | B=-8.41  P=0.001 | People with frequent seizure had a three times decrease in HRQOL mean score as compare to no frequent seizure. |
|  |  | Muche, E.A., et al. [28] | 354 | QOLIE-10 | 19.85±6.91 | P=0.001 | Frequency of seizure had significant correlation with HRQOL mean-score. |
|  |  | Wudu Yesuf.[30] | 340 | QOLIE-31 | 55.6 ±20.9 | B=-4.18  (-6.97, -1.39) | People with frequent seizure had a four times decrease in HRQOL mean score as compare to no frequent seizure |
| Seizure control | Yes/No | Tefera, G. M.[19] | 121 | WHOQOL-BREF | 56.42±10.96 | B=-4.24  P=0.001 | People with uncontrolled seizure had a four times decrease in HRQOL mean score as compare to no frequent seizure |
|  |  | Alemu, A., et al[32]. | 423 | WHOQOL-BREF | 56.17±10.01 | OR=2.2  P=0.01 | People with uncontrolled seizure were 2.2 times more likely to have a low HRQOL mean score as compare to controlled seizure. |
|  |  | Kassie AM, et al. [27] | 395 | QOLIE-31 | 58.48±21.22 | B=-11.08  P=0.001 | People with uncontrolled seizure had 11 times decrease in HRQOL mean score as compare to controlled seizure. |
|  |  | Wudu Yesuf.[30] | 340 | QOLIE-31 | 55.6 ±20.9 | B=6.50  (3.66, 9.33) | People with uncontrolled seizure were 6.50 times more likely to have a low HRQOL mean score as compare to controlled seizure. |
| **Perceived stigma** | JPSS-3 | Mesafint et al.[4] | 439 | WHOQOL-BREF | 61±11.6 | B=-2.13  (-2.96, -1.30) | 1 unit increase in JPSS-3 score was associated with 2.13 unite decrease in WHOQOL-BREF health related QOL score. |
|  | KSSF-3 | Addis, B., et al.[23] | 370 | QOLIE-31 | 55.81±14 | B=-1.11  B=0.001 | 1 unit increase in KSSF-3 score was associated with 1.11 unite decrease in WHOQOL-BREF health related QOL score. |
|  | rESS-3 | Guday, E., et al. [26] | 462 | WHOQOL-BREF | 57.2±12.3 | B=-4.73  P=0.01 | 1 unit increase in rESS-3 score was associated with 4.73 unite decrease in WHOQOL-BREF health related QOL score. |
|  | KSSF-3 | Tegegne, M.T.[20] | 415 | WHOQOL-BREF | 56.36±13.37 | OR=2.14  (1.24, 3.67) | 1 unit increase in KSSF-3 score was associated with unite 2.14 times increase in WHOQOL-BREF health related QOL score. |
|  | Unclear | Abadiga et al.[18] | 392 | WHOQOL-BREF | 60.47±23.07 | B=-9.73  P=0.001 | 1 unit increase in KSSF-3 score was associated with 9.73 unite decrease in WHOQOL-BREF health related QOL score. |
|  | rESS-3 | Wudu Yesuf.[30] | 340 | QOLIE-31 | 55.6 ±20.9 | B=-3.62  (-6.30, -0.94) | 1 unit increase in rESS-3 score was associated with 3.62 unite decrease in QOLIE-31 health related QOL score. |
| **AED adherence** | MARS-5 | Mesafint et al.[4] | 439 | WHOQOL-BREF | 61±11.6 | B=1.24  (1.10, 1.30) | 1 unit increase in MARS-5 score was associated with 1.24 unite increase in WHOQOL-BREF health related QOL score. |
|  | MARS-10 | Guday, E., et al. [26] | 462 | WHOQOL-BREF | 57.2±12.3 | B=-0.06  P=0.01 | 1 unit increase in MARS-10 score was associated with 0.06 unite decrease in WHOQOL-BREF health related QOL score. |
|  | MARS-5 | Tegegne, M.T.[20] | 415 | WHOQOL-BREF | 56.36±13.37 | OR=1.12  (0.63, 2.01) | People with AED adherence were 1.12 times more likely to have an increased HRQOL mean score as compare to non-adhered. |
| **Drug side effect** | LAEP | Mesafint et al.[4] | 439 | WHOQOL-BREF | 61±11.6 | B=-0.32  (-0.38, -0.26) | 1 unit increase in LAEP score scale was associated with 0.32 unite decrease in WHOQOL-BREF health related QOL score. |
|  | LAEP | Addis, B., et al.[23] | 370 | QOLIE-31 | 55.81±14 | B=-7.43  P=0.002 | 1 unit increase in LAEP score scale was associated with 7.43 unite decrease in QOLIE-31 health related QOL score |
|  | unclear | Abadiga et al.[18] | 392 | WHOQOL-BREF | 60.47±23.07 | B=-10.86  P=0.001 | Drug adverse effect was associated 10.86 decrease in WHOQOL-BREF health related QOL score |
| **Anxiety** | HADS-A | Mesafint et al.[4] | 439 | WHOQOL-BREF | 61±11.6 | B=-1.91  (-2.95, -0.86) | 1 unit increase in HADS-A scoring scale was associated with 1.91 unite decrease in WHOQOL-BREF health related QOL score. |
|  | HADS-A | Addis, B., et al.[23] | 370 | QOLIE-31 | 55.81±14 | B=-2.24  P= 0.001 | 1 unit increase in HADS-A scoring scale was associated with 2.24 unite decrease in QOLIE-31 health related QOL score |
|  | HADS-A | Minwuyelet F, et al.[21] | 402 | WHOQOL-BREF | 53.47±18.42 | OR=3.63(2.55, 8.42) | People with anxiety were 3.63 times more likely to have decrease HRQOL mean score as compare anxiety free. |
|  | HADS-A | Guday, E., et al. [26] | 462 | WHOQOL-BREF | 57.2±12.3 | B=-8.85  P=0.01 | 1 unit increase in HADS-A scoring scale was associated with 2.24 unite decrease in WHOQOL-BREF health related QOL score. |
|  | HADS-A | Tegegne, M.T.[20] | 415 | WHOQOL-BREF | 56.36±13.37 | OR=4.49(2.39, 8.44) | People with anxiety were 4.49 times more likely to have decrease HRQOL mean score as compare anxiety free. Hence, more likely to have poor health-related quality of life. |
|  | HADS-A | Wudu Yesuf.[30] | 340 | QOLIE-31 | 55.6 ±20.9 | B=-6.79  (-9.26, -4.32) | 1 unit increase in HADS-A scoring scale was associated with 6.79 unite decrease in QOLIE-31health related QOL score. |
| **Depression** | HADS-D | Mesafint et al.[4] | 439 | WHOQOL-BREF | 61±11.6 | B=-3.9  (-4.6, -2.55) | 1 unit increase in HADS-D scoring scale was associated with 3.9 unite decrease in QOLIE WHOQOL-BREF health related QOL score. |
|  | HADS-D | Addis, B., et al.[23] | 370 | QOLIE-31 | 55.81±14 | B=-2.03  P= 0.001 | 1 unit increase in HADS-D scoring scale was associated with 2.03 unite decrease in QOLIE-31 health related QOL score. |
|  | HADS-D | Minwuyelet F, et al.[21] | 402 | WHOQOL-BREF | 53.47±18.42 | OR=3.85(2.16, 6.82) | People with depression were 3.85 times more likely to have decrease HRQOL mean score as compare depression free. Hence, more likely to have poor health-related quality of life. |
|  | HADS-D | Guday, E., et al. [26] | 462 | WHOQOL-BREF | 57.2±12.3 | B=-11.82  P=0.01 | 1 unit increase in HADS-D scoring scale was associated with 11.82 unite decrease in WHOQOL-BREF health related QOL score. |
|  | HADS-D | Tegegne, M.T.[20] | 415 | WHOQOL-BREF | 56.36±13.37 | OR=9.62(4.86, 19.05) | People with depression were 9.62 times more likely to have decrease HRQOL mean score as compare depression free. Hence, more likely to have poor health-related quality of life. |
|  | HADS-D | Wudu Yesuf.[30] | 340 | QOLIE-31 | 55.6 ±20.9 | B=-7.36  (-10.16, -4.55) | 1 unit increase in HADS-D scoring scale was associated with 7.36 unite decrease in WHOQOL QOLIE-31 health related QOL score. |
| Social support | OSSS | Mesafint et al.[4] | 439 | WHOQOL-BREF | 61±11.6 | B=-2.51  (-3.62, -1.40) | 1 unit increase in OSSS scoring scale was associated with 2.51 unite decrease in WHOQOL-BREF health related QOL score. |
|  | OSSS | Addis, B., et al.[23] | 370 | QOLIE-31 | 55.81±14 | B=2.10  P=0.03 | 1 unit increase in OSSS scoring scale was associated with 2.10 unite increase in QOLIE-31 health related QOL score. |
|  | OSSS | Stotaw et al.[22] | 384 | WHOQOL-BREF | 51.98±10.08 | OR=9.28  P=0.04 | People with social support were 9.28 times more likely to have increase HRQOL mean score as compare no support. Hence, more likely to have good health-related quality of life. |
|  | OSSS | Guday, E., et al. [26] | 462 | WHOQOL-BREF | 57.2±12.3 | B=-10.20  P=0.001 | 1 unit increase in OSSS scoring scale was associated with 10.20 unite decrease in WHOQOL-BREF health related QOL score. |
|  | OSSS | Tsigebrhan, R., et al.[29] | 237 | QOLIE-10 | 69.7±19.3 | B=-9.66  (-16.52, -2.81) | 1 unit increase in OSSS scoring scale was associated with 9.66 unite decrease in QOLIE-10 health related QOL score. |
|  | OSSS | Alemu, A., et al[32]. | 423 | WHOQOL-BREF | 56.17±10.01 | OR= 3.7  P=0.001 | People with social support were 3.7 times more likely to have increase HRQOL mean score as compare no support. Hence, more likely to have good health-related quality of life. |
|  | OSSS | Wudu Yesuf.[30] | 340 | QOLIE-31 | 55.6 ±20.9 | B=0.34  (0.27,0.40) | 1 unit increase in OSSS scoring scale was associated with 0.34 unite increase in QOLIE-31 health related QOL score. |
| **Comorbidity** | Yes/No | Tefera, G. M.[19] | 121 | WHOQOL-BREF | 56.42±10.96 | B=-5.62  P=0.001 | People with comorbidity had 5.62 times decrease HRQOL mean score as compare no comorbidity. Hence, more likely to have poor health-related quality of life. |
|  | Yes/No | Stotaw et al.[22] | 384 | WHOQOL-BREF | 51.98±10.08 | OR=3.57  P=0.001 | People with no comorbidity were 3.57 times more likely to have increase HRQOL mean score as compare to having comorbidity. Hence, more likely to have good health-related quality of life. |
|  | Yes/No | Guday, E., et al. [26] | 462 | WHOQOL-BREF | 57.2±12.3 | B=-4.03  P=0.001 | People with comorbidity had 4.03 times decrease HRQOL mean score as compare no comorbidity. Hence, more likely to have poor health-related quality of life. |
|  | Yes/No | Tsigebrhan, R., et al.[29] | 237 | QOLIE-10 | 69.7±19.3 | B=-13.27  (-23.28, -3.26) | People with comorbidity had 13.27 times decrease HRQOL mean score as compare no comorbidity. Hence, more likely to have poor health-related quality of life. |
|  | Yes/No | Abadiga et al.[18] | 392 | WHOQOL-BREF | 60.47±23.07 | B=-12.99  P=0.001 | People with comorbidity had 12.99 times decrease HRQOL mean score as compare no comorbidity. Hence, more likely to have poor health-related quality of life. |
|  | Yes/No | Kassie AM, et al. [27] | 395 | QOLIE-31 | 58.48±21.22 | B=-9.35  P=0.001 | People with comorbidity had 9.35 times decrease HRQOL mean score as compare no comorbidity. Hence, more likely to have poor health-related quality of life. |
|  | Yes/No | Muche, E.A., et al. [28] | 354 | QOLIE-10 | 19.85±6.91 | P=0.001 | Comorbidity associated with HRQOL |

Note: **OSSS**: Oslo Social support Scale, **JPSS-3**: three-item Jacoby perceived stigma scale, **MARS-5**: Medication Adherence Reporting Scale-5, **LAEP**: Liverpool Adverse Events Profile, **KSSE-3:** The Kilif Stigma Scale of Epilepsy, **HADS**: Hospital Anxiety and Depression Scale, **rESS-3**: the three item revised version of the Epilepsy Stigma Scale
