# Supplementary material for: Health-related quality of life and its predictors among epilepsy patients in Ethiopia: Systematic review and meta-analysis
Source: PLoS One. 2025 Jun 3;20(6):e0324363. doi: 10.1371/journal.pone.0324363 (PMC12132937; doi:10.1371/journal.pone.0324363)
Supplement: S3 Table — (DOCX) [file pone.0324363.s006.docx]

**S2 Table:** All studies identified in the literature search, including those that were excluded from the analyses.

| S/N | Author/s(reference | Title | DOI | Included/excluded | Reason for exclusion | Published or unpublished | URL if unpublished |
| --- | --- | --- | --- | --- | --- | --- | --- |
| 1 | Mesafint, G., et al.[1] | *Quality of life and associated factors among patients with epilepsy attending outpatient department of Saint Amanuel Mental Specialized Hospital, Addis Ababa, Ethiopia, 2019.* | <https://doi.org/10.2147/JMDH.S284958> | Included |  | Published | N/A |
| 2 | Guday, E., et al.[2] | *Quality of life and its associated factors among epileptic patients on treatment follow up in North Shoa administration, Amhara National State, Ethiopia.* | <https://doi.org/10.1101/2022.12.28.22284016> | Included |  | Published | N/A |
| 3 | Ddddd.[3] | *Health related quality of life and associated factors among adult patients with epilepsy attending at Mizan Tepi University Teaching Hospital, South West Ethiopia.* | <https://doi.org/10.1136/bmjopen-2023-079165> | Included |  | Published | N/A |
| 4 | Addis, B., A. Minyihun, and A.Y. Aschalew.[4] | *Health-related quality of life and associated factors among patients with epilepsy at the University of Gondar comprehensive specialized hospital, northwest Ethiopia.* | <https://doi.org/10.1007/s11136-020-02666-4> | Included |  | Published | N/A |
| 5 | Kassie, A.M., et al.[5] | *Quality of life and its associated factors among epileptic patients attending public hospitals in North Wollo Zone, Northeast Ethiopia: A cross-sectional study.* | <https://doi.org/10.1371/journal.pone.0247336> | Included |  | Published | N/A |
| 6 | Teshome, Y., et al.[6] | *Level of Acceptance of Illness and Its Association with Quality of Life among Patients with Epilepsy in North Shewa, Ethiopia.* | <https://doi.org/10.1155/2022/1142215> | Included |  | Published | N/A |
| 7 | Minwuyelet, F., et al.[7] | *Quality of life and associated factors among patients with epilepsy at specialized hospitals, Northwest Ethiopia* | <https://doi.org/10.1371/journal.pone.0262814> | Included |  | Published | N/A |
| 8 | Abadiga, M., et al.[8] | *Health-related quality of life and associated factors among epileptic patients on treatment follow up at public hospitals of Wollega zones, Ethiopia.* | <https://doi.org/10.1186/s13104-019-4720-3> | Included |  | Published | N/A |
| 9 | Alemu, A., et al.[9] | *Health-related quality of life and associated factors among adult patients with epilepsy in public hospitals of Wolaita zone, southern Ethiopia. An embedded mixed method study.* | <https://doi.org/10.1016/j.yebeh.2023.109316> | Included |  | Published | N/A |
| 10 | Gebre, A.K. and A. Haylay[10] | *Sociodemographic, clinical variables, and quality of life in patients with epilepsy in Mekelle City, Northern Ethiopia.* | <https://doi.org/10.1155/2018/7593573> | Included |  | Published | N/A |
| 11 | Shiferaw, D. and E. Hailu,[11] | *Quality of life assessment among adult epileptic patients taking follow up care at Jimma University Medical Center, Jimma, South West Ethiopia: using quality of life in epilepsy Inventory31instrument.* | <https://doi.org/10.1371/journal.pone.0262814> | Included |  | Published | N/A |
| 12 | Muche, E.A., M.B. Ayalew, and O.A. Abdela.[12] | *Assessment of quality of life of epileptic patients in Ethiopia.* | <https://doi.org/10.1155/2020/8714768> | Included |  | Published | N/A |
| 13 | Stotaw, A.S., et al.[13] | *Health-related quality of life and its predictors among people living with epilepsy at Dessie Referral Hospital, Amhara, Ethiopia: A cross-sectional study.* | <https://doi.org/10.1177/20503121221129146> | Included |  | Published | N/A |
| 14 | Tefera, G.M., W.A. Megersa, and D.A. Gadisa.[14] | *Health-related quality of life and its determinants among ambulatory patients with epilepsy at Ambo General Hospital, Ethiopia: Using WHOQOL-BREF.* | <https://doi.org/10.1371/journal.pone.0227858> | Included |  | Published | N/A |
| 15 | Tegegne, M.T., et al.[15] | *Assessment of quality of life and associated factors among people with epilepsy attending at Amanuel Mental Specialized Hospital, Addis Ababa, Ethiopia.* | <http://doi:10.11648/j.sjph.20140205.12> | Included |  | Published | N/A |
| 16 | Tsigebrhan, R., et al.[16] | *Comorbid mental disorders and quality of life of people with epilepsy attending primary health care clinics in rural Ethiopia* | <https://doi.org/10.1371/journal.pone.0238137> | Included |  | Published | N/A |
| 17 | Tamene, F.B., et al.[17] | *Health-related quality of life and associated factors among health care providers in the northwest of Ethiopia: a multicenter cross-sectional study* | <https://doi.org/10.3389/fpubh.2024.1357856> | Excluded | Target population difference | Published | N/A |
| 18 | Biset, G. and A. Woday.[18] | *Epilepsy treatment outcomes in the referral hospitals of northeast Ethiopia.* | <https://doi.org/10.1016/j.eplepsyres.2021.106584> | Excluded | Tittle and outcome of interest difference | Published | N/A |
| 19 | Ahmed, I., et al.[19] | *Self-care practice and associated factors among epileptic patients: a cross-sectional study, Ethiopia.* | <https://doi.org/10.11604/pamj.2023.44.36.31554> | Excluded | Tittle and outcome of interest difference | Published | N/A |
| 20 | Solomon, Y., et al.[20] | *Prevalence of anti-seizure medication nonadherence and its associated factors, among people with epilepsy in North Shewa, Ethiopia* | <https://doi.org/10.1016/j.yebeh.2023.109301> | Excluded | outcome of interest difference | unpublished | N/A |
| 21 | Ashjazadeh, N., et al.[21] | *Comparison of the health-related quality of life between epileptic patients with partial and generalized seizure.* | Not found |  | Not Ethiopian study | unpublished | N/A |
| 22 | Addis, B., et al.[22] | *Prevalence of depression and associated factors among patients with epilepsy at the University of Gondar Comprehensive Specialized Hospital, Northwest Ethiopia.* | <https://doi.org/10.1371/journal.pone.0257942> | Excluded |  | Published | N/A |
| 23 | Tsegabrhan, H., et al.[23] | *Co-morbidity of depression and epilepsy in Jimma University specialized hospital, Southwest Ethiopia.* | <https://doi.org/10.4103/0028-3886.149391> | Excluded | Outcome of interest difference | Published | N/A |
| 24 | Asnakew, S., et al.[24] | *Cognitive adverse effects of epilepsy and its predictors attending outpatient department of South Gondar zone hospitals, Amhara Region, Ethiopia.* | <https://doi.org/10.1371/journal.pone.0278908> | Excluded | Outcome of interest difference | Published | N/A |
| 25 | Kováts, D., et al.[25] | *Assessment of health related quality of life among epileptic patients in the context of coping strategies and subjective disease perception.* | <https://doi.org/10.18071/isz.71.0184> | Excluded | Not Ethiopian study | Published | N/A |
| 26 | Flewelling, K.D., et al.[26] | *Correlates of health-related quality of life in youth with psychogenic non-epileptic seizures.* | <https://doi.org/10.1016/j.seizure.2020.09.030> | Excluded | Target population difference | Published | N/A |
| 27 | Beyene, Y.B., et al.[27] | *Drug therapy problems and predicting factors among ambulatory epileptic patients in Jimma Medical Center, Southwest Ethiopia.* | <https://doi.org/10.1371/journal.pone.0267673> | Excluded | Outcome of interest difference | Published | N/A |
| 28 | Yerdelen D, et al. [28] | *Health related quality of life in patients admitted for video-electroencephalography monitoring diagnosed with epilepsy or psychogenic non-epileptic seizures.* | <https://doi.org/10.17712/nsj.2016.1.2015595> | Excluded | Not Ethiopian study | Published | N/A |
| 29 | Widjaja, E., et al.[29] | *Trajectory of Health-Related Quality of Life After Pediatric Epilepsy Surgery.* | <https://doi.org/10.1001/jamanetworkopen.2023.4858> | Excluded | Outcome of interest difference | Published | N/A |
| 30 | Seid, J.,et al. [30] | *Prevalence and associated factors of anxiety disorder symptoms among people with epilepsy in Mekelle, Ethiopia.* | <https://doi.org/10.1002/nop2.1198> | Excluded | Outcome of interest difference | Published | N/A |
| 31 | Hamedi-Shahraki, S., et al.[31] | *Health-related quality of life and medication adherence in elderly patients with epilepsy.* | <https://doi.org/10.5603/pjnns.a2019.0008> | Excluded |  | Published | N/A |
| 32 | Adal, H.D.,et al.[32] | *Seizure control status and associated factors among pediatric epileptic patients at a neurologic outpatient clinic in Ethiopia* | <https://doi.org/10.1371/journal.pone.0259079> | Excluded | Target population difference | Published | N/A |
| 33 | Eskandrani, A., et al.[33] | *Health related quality of life in a Saudi population of patients with epilepsy.* | <https://doi.org/10.17712/nsj.2023.4.20230026> | Excluded | Study area difference | Published | N/A |
| 34 | Altwijri, R.M.,et al. [34] | *Quality of life among epileptic patients in Qassim Region, KSA.* | <https://doi.org/10.17712/nsj.2021.1.20200044> | Excluded | Study area difference | Published | N/A |
| 35 | Shumet, S., et al.[35] | *Antiepileptic Drug Adherence and Its Associated Factors among Epilepsy Patients on Follow-ups at Amanuel Mental Specialized Hospital, Ethiopia.* | <https://doi.org/10.4314/ejhs.v32i5.6> | Excluded | Outcome of interest difference | Published | N/A |
| 36 | Chiang, J.A., et al.[36] | *Neighborhood disadvantage and health-related quality of life in pediatric epilepsy.* | <https://doi.org/10.1016/j.yebeh.2023.109171> | Excluded | Target population difference | Published | N/A |
| 37 | Winter, Y., et al.[37] | *Health-related quality of life in patients with poststroke epilepsy* | <https://doi.org/10.1016/j.yebeh.2017.12.037> | Excluded | Study area difference | Published | N/A |
| 38 | Mohammed, H., et al.[38] | *Adherence to anti-seizure medications and associated factors among children with epilepsy at tertiary Hospital in Southwest Ethiopia: a cross-sectional study* | <https://doi.org/10.1186/s12883-022-02842-8> | Excluded | Outcome of interest difference | published | N/A |
| 39 | Nabukenya, A.M., et al.[39] | *Health-related quality of life in epilepsy patients receiving anti-epileptic drugs at National Referral Hospitals in Uganda: a cross-sectional study.* | <https://doi.org/10.1186/1477-7525-12-49> | Excluded | Study area difference | published | N/A |
| 40 | Alsaadi, T., et al.[40] | *Potential factors impacting health-related quality of life among patients with epilepsy: Results from the United Arab Emirates.* Seizure. | <https://doi.org/10.1016/j.seizure.2017.10.017> | Excluded | Study area difference | published | N/A |
| 41 | Victor Menezes Sousa, A., et al.[41] | *Potential factors impacting health-related quality of life among patients with epilepsy.* | <https://doi.org/10.1016/j.yebeh.2021.107969> | Excluded | Study area difference | published | N/A |
| 42 | Victor Menezes Sousa, A., et al.[41] | *Validation of the Health-Related Quality of Life in Childhood Epilepsy Questionnaire (QOLCE-55) for Brazilian Portuguese.* | <https://doi.org/10.1016/j.yebeh.2018.05.009> | Excluded | Target population difference | published | N/A |
| 43 | Salinsky, M., et al.[42] | *Health-related quality of life in Veterans with epileptic and psychogenic nonepileptic seizures.* | <https://doi.org/10.1016/j.yebeh.2019.02.010> | Excluded | Study area difference | published | N/A |
| 44 | Kverneland, M., et al.[43] | *Health-related quality of life in adults with drug-resistant focal epilepsy treated with modified Atkins diet in a randomized clinical trial.* | <https://doi.org/10.1111/epi.17585> | Excluded | Study area difference | published | N/A |
| 45 | Rawlings, G.H.,et al.[44] | *Predictors of health-related quality of life in patients with epilepsy and psychogenic nonepileptic seizures.* | <https://doi.org/10.1016/j.yebeh.2016.10.035> | Excluded | Study area difference | published | N/A |
| 46 | Jain, P., et al.[45] | *Seizure freedom improves health-related quality of life after epilepsy surgery in children.* | <https://doi.org/10.1111/dmcn.14390> | Excluded | Target population difference | published | N/A |
| 47 | An, O., et al.[46] | *Comparative assessment of health-related quality of life with and without anticonvulsant therapy in patients with childhood epilepsy with centrotemporal spikes.* | <https://doi.org/10.1177/03000605211039805> | Excluded | Target population difference | published | N/A |
| 48 | Chiang, S., et al.[47] | *Seizure detection devices and health-related quality of life: A patient- and caregiver-centered evaluation.* | <https://doi.org/10.1016/j.yebeh.2020.106963> | Excluded | Outcome of interest difference | published | N/A |
| 49 | Edelvik, A., et al.[48] | *Health-related quality of life and emotional well-being after epilepsy surgery: A prospective, controlled, long-term follow-up.* | <https://doi.org/10.1111/epi.13874> | Excluded | Study area difference | published | N/A |
| 50 | Lotfinia, M., et al.[49] | *Health-related quality of life after epilepsy surgery: A prospective, controlled follow-up on the Iranian population.* | <https://doi.org/10.1038/s41598-019-44442-6> | Excluded | Study area difference | published | N/A |
| 51 | Lin, P.T., et al.[50] | *Social functioning and health-related quality of life trajectories in people with epilepsy after epilepsy surgery.* | <https://doi.org/10.1016/j.yebeh.2019.106849> | Excluded | Study area difference | published | N/A |
| 52 | Friedman, D.E., S. Islam, and A.B.[51] | Ettinger, *Health-related quality of life among people with epilepsy with mild seizure-related head injuries.* | <https://doi.org/10.1016/j.yebeh.2013.02.009> | Excluded | Target population difference, Study area difference | published | N/A |
| 53 | Wester, V., et al.[52] | *Good Days and Bad Days: Measuring Health-Related Quality of Life in People With Epilepsy* | <https://doi.org/10.1016/j.jval.2021.05.001> | Excluded | Study area difference | published | N/A |
| 54 | Ogundare, T., eta al.[53] | *Quality of life among patients with epilepsy in Nigeria* | <https://doi.org/10.1007/s11136-020-02643-x> | Excluded | Study area difference | published | N/A |
| 55 | Guilfoyle, S.M., et al.[54] | Guilfoyle, S.M., et al., *Quality of life improves with integrated behavioral health services in pediatric new-onset epilepsy* | <https://doi.org/10.1016/j.yebeh.2019.04.017> | Excluded | Target population difference, Study area difference | published | N/A |
| 56 | Adebayo, P.B., et al.[55] | *Seizure severity and health-related quality of life of adult Nigerian patients with epilepsy.* | <https://doi.org/10.1111/ane.12146> | Excluded | Study area difference | published | N/A |
| 57 | Chou, C.C., et al.[56] | *Long-term health-related quality of life in drug-resistant temporal lobe epilepsy after anterior temporal lobectomy* | <https://doi.org/10.1684/epd.2015.0744> | Excluded | Study area difference | published | N/A |
| 58 | Iwuozo, E.U., et al.[57] | *Determinants of Health-related Quality of Life in Persons with Epilepsy Seen at a Tertiary Hospital in North Western Nigeria.* | Not found | Excluded | Study area difference | published | N/A |
| 59 | Drulovic, J., et al.[58] | *The impact of the comorbid seizure/epilepsy on the health related quality of life in people with multiple sclerosis: an international multicentric study* | <https://doi.org/10.3389/fimmu.2023.1284031> | Excluded | Target population difference, Study area difference | published | N/A |
| 60 | Yadegary, M.A., et al.[59] | *The effect of self-management training on health-related quality of life in patients with epilepsy.* | <https://doi.org/10.1016/j.yebeh.2015.04.051> | Excluded | The tool is not clear | published | N/A |
| 61 | Lua, P.L. and W.S. Neni.[60] | *Health-related quality of life improvement via telemedicine for epilepsy* | <https://doi.org/10.1007/s11136-013-0352-6> | Excluded | The tool is not clear  Study area difference | published | N/A |
| 62 | Conway, L., E. Widjaja, and M.L. Smith.[61] | , *Impact of resective epilepsy surgery on health-related quality of life in children with and without low intellectual ability.* | <https://doi.org/10.1016/j.yebeh.2018.03.036> | Excluded | Target population difference | published | N/A |
| 63 | Zashikhina, A. and B. Hagglof.[62] | *Health-related quality of life in adolescents with chronic physical illness in northern Russia: a cross-sectional study.* | <https://doi.org/10.1186/1477-7525-12-12> | Excluded | Target population difference | published | N/A |
| 64 | Lee, S.A.,et al.[63] | *Felt stigma in seizure-free persons with epilepsy: Associated factors and its impact on health-related quality of life.* | <https://doi.org/10.1016/j.yebeh.2021.108186> | Excluded | Study area difference  The tool is not clear | published | N/A |
| 65 | Brandt, C., et al.[64] | *Health-related quality of life in double-blind Phase III studies of brivaracetam as adjunctive therapy of focal seizures: A pooled, post-hoc analysis* | <https://doi.org/10.1016/j.yebeh.2016.11.031> | Excluded | Study area difference | published | N/A |
| 66 | Puka, K., K.N. Speechley, and M.A. Ferro.[65] | *Convulsive status epilepticus in children recently diagnosed with epilepsy and long-term health-related quality of life* | <https://doi.org/10.1016/j.seizure.2020.05.025> | Excluded | Target population difference | published | N/A |
| 67 | De la Loge, C., et al.[66] | *PatientsLikeMe® Online Epilepsy Community: Patient characteristics and predictors of poor health-related quality of life.* | <https://doi.org/10.1016/j.yebeh.2016.07.035> | Excluded | Study area difference | published | N/A |
| 68 | Endermann, M.[67] | *Predictors of health-related and global quality of life among young adults with difficult-to-treat epilepsy and mild intellectual disability.* | <https://doi.org/10.1016/j.yebeh.2012.12.002> | Excluded | Study area difference  The tool is not clear | published | N/A |
| 69 | Jadhav, P.M., et al.[68] | *Assessment and comparison of health-related quality-of-life (HRQOL) in patients with epilepsy in India.* | <https://doi.org/10.1016/j.yebeh.2012.12.027> | Excluded | Study area difference | published | N/A |
| 70 | Gao, L., et al.[69] | *Reliability and validity of QOLIE-10 in measuring health-related quality of life (HRQoL) in Chinese epilepsy patients.* | <https://doi.org/10.1016/j.eplepsyres.2014.01.007> | Excluded | Study area difference | published | N/A |
| 71 | Modi, A.C., et al.[70] | *Validation of the PedsQL Epilepsy Module: A pediatric epilepsy-specific health-related quality of life measure* | <https://doi.org/10.1111/epi.13875> | Excluded | Target population difference | published | N/A |
| 72 | Losada-Camacho, M., et al.[71] | *Impact of a pharmaceutical care programme on health-related quality of life among women with epilepsy:* | <https://doi.org/10.1186/s12955-014-0162-8> | Excluded | Target population difference | published | N/A |
| 73 | Barranco-Camargo, L.A., et al.[72] | *[Validity and reliability of the QOLIE-10 instrument for assessing health related quality of life in epilepsy of refractory epilepsy adult patients at a Colombian neurological center* | <https://doi.org/10.33588/rn.6912.2019273> | Excluded | Study area difference | published | N/A |
| 74 | Péntek, M., et al.[73] | *Survey of adults living with epilepsy in Hungary: health-related quality of life and costs* | Not found | Excluded | Study area difference | published | N/A |
| 75 | Al Hayek, A.A., et al.[74] | *Factors associated with health-related quality of life among Saudi patients with type 2 diabetes mellitus: a cross-sectional survey.* | <http://dx.doi.org/10.4093/dmj.2014.38.3.220> | Excluded | Target population and Outcome of interest difference | published | N/A |
| 76 | Friedman, D.E.,et al.[75] | *Health-related quality of life among people with epilepsy with mild seizure-related head injuries.* | <https://doi.org/10.1016/j.yebeh.2013.02.009> | Excluded | Study area difference | published | N/A |
| 77 | Adebayo, P., et al.[76] | *Seizure severity and health‐related quality of life of adult Nigerian patients with epilepsy.* | <https://doi.org/10.1111/ane.12146> | Excluded | Study area difference | published | N/A |
| 78 | Momeni, M., et al.[77] | *Health-related quality of life and related factors in children and adolescents with epilepsy in Iran.* | *DOI:*10.1097/JNN.0000000000000173 | Excluded | Study area and Target population difference | published | N/A |
| 79 | Aronu, A., et al.[78] | *Health-related quality of life in children and adolescents with epilepsy in Enugu: Need for targeted intervention* | DOI:10.4103/njcp.njcp_457_20 | Excluded | Target population difference | published | N/A |
| 80 | Fawale, M.B.,et al.[79] | *Effects of seizure severity and seizure freedom on the health-related quality of life of an African population of people with epilepsy* | <https://doi.org/10.1016/j.yebeh.2013.12.026> | Excluded | Outcome of interest difference | published | N/A |
| 81 | Radović, N.I., et al.[80] | *Health-related quality of life in adolescents with epilepsy in Montenegro* | <https://doi.org/10.1016/j.yebeh.2017.07.009> | Excluded | Target population difference | published | N/A |

Note: N/A; Note applicable in which no study found unpublished

References

1. Mesafint, G., et al., *Quality of life and associated factors among patients with epilepsy attending outpatient department of Saint Amanuel Mental Specialized Hospital, Addis Ababa, Ethiopia, 2019.* Journal of Multidisciplinary Healthcare, 2020: p. 2021-2030.

2. Guday, E., et al., *Quality of life and its associated factors among epileptic patients on treatment follow up in North Shoa administration, Amhara National State, Ethiopia.* medRxiv, 2022: p. 2022.12. 28.22284016.

3. Yesuf, W., et al., *Health-related quality of life in epilepsy and its associated factors among adult patients with epilepsy attending Mizan Tepi University Teaching Hospital, Southwest Ethiopia: a cross-sectional study.* 2024. **14**(1): p. e079165.

4. Addis, B., A. Minyihun, and A.Y. Aschalew, *Health-related quality of life and associated factors among patients with epilepsy at the University of Gondar comprehensive specialized hospital, northwest Ethiopia.* Quality of Life Research, 2021. **30**: p. 729-736.

5. Kassie, A.M., et al., *Quality of life and its associated factors among epileptic patients attending public hospitals in North Wollo Zone, Northeast Ethiopia: A cross-sectional study.* PloS one, 2021. **16**(2): p. e0247336.

6. Teshome, Y., et al., *Level of Acceptance of Illness and Its Association with Quality of Life among Patients with Epilepsy in North Shewa, Ethiopia.* Behavioural Neurology, 2022. **2022**(1): p. 1142215.

7. Minwuyelet, F., et al., *Quality of life and associated factors among patients with epilepsy at specialized hospitals, Northwest Ethiopia; 2019.* PLoS One, 2022. **17**(1): p. e0262814.

8. Abadiga, M., et al., *Health-related quality of life and associated factors among epileptic patients on treatment follow up at public hospitals of Wollega zones, Ethiopia, 2018.* BMC research notes, 2019. **12**: p. 1-7.

9. Alemu, A., et al., *Health-related quality of life and associated factors among adult patients with epilepsy in public hospitals of Wolaita zone, southern Ethiopia. An embedded mixed method study.* Epilepsy & Behavior, 2023. **145**: p. 109316.

10. Gebre, A.K. and A. Haylay, *Sociodemographic, clinical variables, and quality of life in patients with epilepsy in Mekelle City, Northern Ethiopia.* Behavioural neurology, 2018. **2018**(1): p. 7593573.

11. Shiferaw, D. and E. Hailu, *Quality of life assessment among adult epileptic patients taking follow up care at Jimma University Medical Center, Jimma, South West Ethiopia: using quality of life in epilepsy Inventory31instrument.* Global Journal of medical research, 2018. **18**(3).

12. Muche, E.A., M.B. Ayalew, and O.A. Abdela, *Assessment of quality of life of epileptic patients in Ethiopia.* International Journal of Chronic Diseases, 2020. **2020**(1): p. 8714768.

13. Stotaw, A.S., et al., *Health-related quality of life and its predictors among people living with epilepsy at Dessie Referral Hospital, Amhara, Ethiopia: A cross-sectional study.* SAGE Open Medicine, 2022. **10**: p. 20503121221129146.

14. Tefera, G.M., W.A. Megersa, and D.A. Gadisa, *Health-related quality of life and its determinants among ambulatory patients with epilepsy at Ambo General Hospital, Ethiopia: Using WHOQOL-BREF.* PloS one, 2020. **15**(1): p. e0227858.

15. Tegegne, M.T., et al., *Assessment of quality of life and associated factors among people with epilepsy attending at Amanuel Mental Specialized Hospital, Addis Ababa, Ethiopia.* Science, 2014. **2**(5): p. 268-73.

16. Tsigebrhan, R., et al., *Comorbid mental disorders and quality of life of people with epilepsy attending primary health care clinics in rural Ethiopia.* Plos one, 2021. **16**(1): p. e0238137.

17. Tamene, F.B., et al., *Health-related quality of life and associated factors among health care providers in the northwest of Ethiopia: a multicenter cross-sectional study, 2023.* Front Public Health, 2024. **12**: p. 1357856.

18. Biset, G. and A. Woday, *Epilepsy treatment outcomes in the referral hospitals of northeast Ethiopia.* Epilepsy Res, 2021. **171**: p. 106584.

19. Ahmed, I., et al., *Self-care practice and associated factors among epileptic patients: a cross-sectional study, Ethiopia.* Pan Afr Med J, 2023. **44**: p. 36.

20. Solomon, Y., et al., *Prevalence of anti-seizure medication nonadherence and its associated factors, among people with epilepsy in North Shewa, Ethiopia, 2021.* Epilepsy & Behavior, 2023. **145**: p. 109301.

21. Ashjazadeh, N., et al., *Comparison of the health-related quality of life between epileptic patients with partial and generalized seizure.* Iran J Neurol, 2014. **13**(2): p. 94-100.

22. Addis, B., et al., *Prevalence of depression and associated factors among patients with epilepsy at the University of Gondar Comprehensive Specialized Hospital, Northwest Ethiopia, 2019.* PLoS One, 2021. **16**(10): p. e0257942.

23. Tsegabrhan, H., et al., *Co-morbidity of depression and epilepsy in Jimma University specialized hospital, Southwest Ethiopia.* Neurol India, 2014. **62**(6): p. 649-55.

24. Asnakew, S., et al., *Cognitive adverse effects of epilepsy and its predictors attending outpatient department of South Gondar zone hospitals, Amhara Region, Ethiopia 2020 /2021.* PLoS One, 2022. **17**(12): p. e0278908.

25. Kováts, D., et al., *[Assessment of health related quality of life among epileptic patients in the context of coping strategies and subjective disease perception].* Ideggyogy Sz, 2018. **71**(5-06): p. 184-196.

26. Flewelling, K.D., et al., *Correlates of health-related quality of life in youth with psychogenic non-epileptic seizures.* Seizure, 2020. **83**: p. 203-207.

27. Beyene, Y.B., et al., *Drug therapy problems and predicting factors among ambulatory epileptic patients in Jimma Medical Center, Southwest Ethiopia.* PLoS One, 2022. **17**(4): p. e0267673.

28. Yerdelen, D. and E. Altintas, *Health related quality of life in patients admitted for video-electroencephalography monitoring diagnosed with epilepsy or psychogenic non-epileptic seizures.* Neurosciences (Riyadh), 2016. **21**(1): p. 47-51.

29. Widjaja, E., et al., *Trajectory of Health-Related Quality of Life After Pediatric Epilepsy Surgery.* JAMA Netw Open, 2023. **6**(3): p. e234858.

30. Seid, J., K. Mebrahtu, and F. Andualem, *Prevalence and associated factors of anxiety disorder symptoms among people with epilepsy in Mekelle, Ethiopia, 2019: Institutional-based cross-sectional study.* Nurs Open, 2022. **9**(3): p. 1731-1743.

31. Hamedi-Shahraki, S., et al., *Health-related quality of life and medication adherence in elderly patients with epilepsy.* Neurol Neurochir Pol, 2019. **53**(2): p. 123-130.

32. Adal, H.D., K. Alemu, and E.A. Muche, *Seizure control status and associated factors among pediatric epileptic patients at a neurologic outpatient clinic in Ethiopia.* 2021. **16**(11): p. e0259079.

33. Eskandrani, A., et al., *Health related quality of life in a Saudi population of patients with epilepsy.* Neurosciences (Riyadh), 2023. **28**(4): p. 234-242.

34. Altwijri, R.M., M.S. Aljohani, and H.K. Alshammari, *Quality of life among epileptic patients in Qassim Region, KSA.* Neurosciences (Riyadh), 2021. **26**(1): p. 56-61.

35. Shumet, S., et al., *Antiepileptic Drug Adherence and Its Associated Factors among Epilepsy Patients on Follow-ups at Amanuel Mental Specialized Hospital, Ethiopia.* Ethiop J Health Sci, 2022. **32**(5): p. 913-922.

36. Chiang, J.A., et al., *Neighborhood disadvantage and health-related quality of life in pediatric epilepsy.* Epilepsy Behav, 2023. **142**: p. 109171.

37. Winter, Y., et al., *Health-related quality of life in patients with poststroke epilepsy.* Epilepsy Behav, 2018. **80**: p. 303-306.

38. Mohammed, H., et al., *Adherence to anti-seizure medications and associated factors among children with epilepsy at tertiary Hospital in Southwest Ethiopia: a cross-sectional study.* BMC Neurol, 2022. **22**(1): p. 310.

39. Nabukenya, A.M., et al., *Health-related quality of life in epilepsy patients receiving anti-epileptic drugs at National Referral Hospitals in Uganda: a cross-sectional study.* Health Qual Life Outcomes, 2014. **12**: p. 49.

40. Alsaadi, T., et al., *Potential factors impacting health-related quality of life among patients with epilepsy: Results from the United Arab Emirates.* Seizure, 2017. **53**: p. 13-17.

41. Victor Menezes Sousa, A., et al., *Validation of the Health-Related Quality of Life in Childhood Epilepsy Questionnaire (QOLCE-55) for Brazilian Portuguese.* Epilepsy Behav, 2021. **120**: p. 107969.

42. Salinsky, M., et al., *Health-related quality of life in Veterans with epileptic and psychogenic nonepileptic seizures.* Epilepsy Behav, 2019. **94**: p. 72-77.

43. Kverneland, M., et al., *Health-related quality of life in adults with drug-resistant focal epilepsy treated with modified Atkins diet in a randomized clinical trial.* Epilepsia, 2023. **64**(5): p. e69-e74.

44. Rawlings, G.H., I. Brown, and M. Reuber, *Predictors of health-related quality of life in patients with epilepsy and psychogenic nonepileptic seizures.* Epilepsy Behav, 2017. **68**: p. 153-158.

45. Jain, P., et al., *Seizure freedom improves health-related quality of life after epilepsy surgery in children.* 2020. **62**(5): p. 600-608.

46. An, O., et al., *Comparative assessment of health-related quality of life with and without anticonvulsant therapy in patients with childhood epilepsy with centrotemporal spikes.* J Int Med Res, 2021. **49**(8): p. 3000605211039805.

47. Chiang, S., et al., *Seizure detection devices and health-related quality of life: A patient- and caregiver-centered evaluation.* Epilepsy Behav, 2020. **105**: p. 106963.

48. Edelvik, A., et al., *Health-related quality of life and emotional well-being after epilepsy surgery: A prospective, controlled, long-term follow-up.* Epilepsia, 2017. **58**(10): p. 1706-1715.

49. Lotfinia, M., et al., *Health-related quality of life after epilepsy surgery: A prospective, controlled follow-up on the Iranian population.* Sci Rep, 2019. **9**(1): p. 7875.

50. Lin, P.T., et al., *Social functioning and health-related quality of life trajectories in people with epilepsy after epilepsy surgery.* Epilepsy Behav, 2020. **103**(Pt A): p. 106849.

51. Friedman, D.E., S. Islam, and A.B. Ettinger, *Health-related quality of life among people with epilepsy with mild seizure-related head injuries.* Epilepsy Behav, 2013. **27**(3): p. 492-6.

52. Wester, V., et al., *Good Days and Bad Days: Measuring Health-Related Quality of Life in People With Epilepsy.* Value Health, 2021. **24**(10): p. 1470-1475.

53. Ogundare, T., T.O. Adebowale, and O.A. Okonkwo, *Quality of life among patients with epilepsy in Nigeria: predictors and barriers to routine clinical use of QOLIE-31.* Qual Life Res, 2021. **30**(2): p. 487-496.

54. Guilfoyle, S.M., et al., *Quality of life improves with integrated behavioral health services in pediatric new-onset epilepsy.* Epilepsy Behav, 2019. **96**: p. 57-60.

55. Adebayo, P.B., et al., *Seizure severity and health-related quality of life of adult Nigerian patients with epilepsy.* Acta Neurol Scand, 2014. **129**(2): p. 102-8.

56. Chou, C.C., et al., *Long-term health-related quality of life in drug-resistant temporal lobe epilepsy after anterior temporal lobectomy.* Epileptic Disord, 2015. **17**(2): p. 177-83.

57. Iwuozo, E.U., et al., *Determinants of Health-related Quality of Life in Persons with Epilepsy Seen at a Tertiary Hospital in North Western Nigeria.* West Afr J Med, 2020. **37**(5): p. 475-480.

58. Drulovic, J., et al., *The impact of the comorbid seizure/epilepsy on the health related quality of life in people with multiple sclerosis: an international multicentric study.* Front Immunol, 2023. **14**: p. 1284031.

59. Yadegary, M.A., et al., *The effect of self-management training on health-related quality of life in patients with epilepsy.* Epilepsy Behav, 2015. **50**: p. 108-12.

60. Lua, P.L. and W.S. Neni, *Health-related quality of life improvement via telemedicine for epilepsy: printed versus SMS-based education intervention.* Qual Life Res, 2013. **22**(8): p. 2123-32.

61. Conway, L., E. Widjaja, and M.L. Smith, *Impact of resective epilepsy surgery on health-related quality of life in children with and without low intellectual ability.* Epilepsy Behav, 2018. **83**: p. 131-136.

62. Zashikhina, A. and B. Hagglof, *Health-related quality of life in adolescents with chronic physical illness in northern Russia: a cross-sectional study.* Health Qual Life Outcomes, 2014. **12**: p. 12.

63. Lee, S.A., *Felt stigma in seizure-free persons with epilepsy: Associated factors and its impact on health-related quality of life.* Epilepsy Behav, 2021. **122**: p. 108186.

64. Brandt, C., et al., *Health-related quality of life in double-blind Phase III studies of brivaracetam as adjunctive therapy of focal seizures: A pooled, post-hoc analysis.* Epilepsy Behav, 2017. **69**: p. 80-85.

65. Puka, K., K.N. Speechley, and M.A. Ferro, *Convulsive status epilepticus in children recently diagnosed with epilepsy and long-term health-related quality of life.* Seizure, 2020. **80**: p. 49-52.

66. de la Loge, C., et al., *PatientsLikeMe® Online Epilepsy Community: Patient characteristics and predictors of poor health-related quality of life.* Epilepsy Behav, 2016. **63**: p. 20-28.

67. Endermann, M., *Predictors of health-related and global quality of life among young adults with difficult-to-treat epilepsy and mild intellectual disability.* Epilepsy Behav, 2013. **26**(2): p. 188-95.

68. Jadhav, P.M., et al., *Assessment and comparison of health-related quality-of-life (HRQOL) in patients with epilepsy in India.* Epilepsy Behav, 2013. **27**(1): p. 165-8.

69. Gao, L., et al., *Reliability and validity of QOLIE-10 in measuring health-related quality of life (HRQoL) in Chinese epilepsy patients.* Epilepsy Res, 2014. **108**(3): p. 565-75.

70. Modi, A.C., et al., *Validation of the PedsQL Epilepsy Module: A pediatric epilepsy-specific health-related quality of life measure.* Epilepsia, 2017. **58**(11): p. 1920-1930.

71. Losada-Camacho, M., et al., *Impact of a pharmaceutical care programme on health-related quality of life among women with epilepsy: a randomised controlled trial (IPHIWWE study).* Health Qual Life Outcomes, 2014. **12**: p. 162.

72. Barranco-Camargo, L.A., et al., *[Validity and reliability of the QOLIE-10 instrument for assessing health related quality of life in epilepsy of refractory epilepsy adult patients at a Colombian neurological center].* Rev Neurol, 2019. **69**(12): p. 473-480.

73. Péntek, M., et al., *[Survey of adults living with epilepsy in Hungary: health-related quality of life and costs].* Ideggyogy Sz, 2013. **66**(7-8): p. 251-61.

74. Al Hayek, A.A., et al., *Factors associated with health-related quality of life among Saudi patients with type 2 diabetes mellitus: a cross-sectional survey.* Diabetes & metabolism journal, 2014. **38**(3): p. 220.

75. Friedman, D.E., S. Islam, and A.B. Ettinger, *Health-related quality of life among people with epilepsy with mild seizure-related head injuries.* Epilepsy & Behavior, 2013. **27**(3): p. 492-496.

76. Adebayo, P., et al., *Seizure severity and health‐related quality of life of adult N igerian patients with epilepsy.* Acta neurologica Scandinavica, 2014. **129**(2): p. 102-108.

77. Momeni, M., et al., *Health-related quality of life and related factors in children and adolescents with epilepsy in Iran.* Journal of Neuroscience Nursing, 2015. **47**(6): p. 340-345.

78. Aronu, A., et al., *Health-related quality of life in children and adolescents with epilepsy in Enugu: Need for targeted intervention.* Nigerian journal of clinical practice, 2021. **24**(4): p. 517-524.

79. Fawale, M.B., M.O. Owolabi, and A. Ogunniyi, *Effects of seizure severity and seizure freedom on the health-related quality of life of an African population of people with epilepsy.* Epilepsy & Behavior, 2014. **32**: p. 9-14.

80. Radović, N.I., et al., *Health-related quality of life in adolescents with epilepsy in Montenegro.* Epilepsy & Behavior, 2017. **76**: p. 105-109.
