# Supplementary material for: Health-related quality of life and its predictors among epilepsy patients in Ethiopia: Systematic review and meta-analysis
Source: PLoS One. 2025 Jun 3;20(6):e0324363. doi: 10.1371/journal.pone.0324363 (PMC12132937; doi:10.1371/journal.pone.0324363)
Supplement: S4 Table — (DOCX) [file pone.0324363.s007.docx]

**S3 Table:** A table of all data extracted from the primary research sources for the systematic review and/or meta-analysis

| s/n | Author/s | Publication year | Region | Sample size | Mean score HRQOL | DS | Response rate | Tool used | Study design | Model/regression | Name of data extractor | Date of data extraction | Eligibility confirmation |
| --- | --- | --- | --- | --- | --- | --- | --- | --- | --- | --- | --- | --- | --- |
| 1 | Abadiga et al. | 2019 | Oromia | 392 | 60.47 | 23.07 | 97.5 | WHOQOL-BREF | Cross-sectional | Linear | HKA, MCA and AWA | Nov. 2023 to Decr.2023. | Eligible |
| 2 | Tefera, G. M. | 2020 | Oromia | 121 | 56.42 | 10.96 | 100 | WHOQOL-BREF | Cross-sectional | Linear | HKA, MCA and AWA | Nov. 2023 to Decr.2023. | Eligible |
| 3 | Tegegne, M.T. | 2014 | Oromia | 415 | 56.36 | 13.37 | 98 | WHOQOL-BREF | Cross-sectional | Logistic | HKA, MCA and AWA | Nov. 2023 to Decr.2023. | Eligible |
| 4 | Minwuyelet F, et al. | 2022 | Amhara | 402 | 53.47 | 18.42 | 96.5 | WHOQOL-BREF | Cross-sectional | logistic | HKA, MCA and AWA | Nov. 2023 to Decr.2023. | Eligible |
| 5 | Stotaw et al. | 2022 | Amhara | 384 | 51.98 | 10.08 | 99.7 | WHOQOL-BREF | Cross-sectional | Linear | HKA, MCA and AWA | Nov. 2023 to Decr.2023. | Eligible |
| 6 | Addis, B., et al. | 2020 | Amhara | 370 | 55.81 | 14 | 98.4 | QOLIE-31 | Cross-sectional | Linear | HKA, MCA and AWA | Nov. 2023 to Decr.2023. | Eligible |
| 7 | Gebre, A.K., et al. | 2018 | Tigray | 175 | 77.97 | 20.78 | 100 | QOLIE-31 | Cross-sectional | Linear | HKA, MCA and AWA | Nov. 2023 to Decr.2023. | Eligible |
| 8 | Minyihun,A.,et al. | 2022 | Amhara | 96 | 79.14 | 25.46 | 97.96 | QOLIE-31 | Cross-sectional | Linear | HKA, MCA and AWA | Nov. 2023 to Decr.2023. | Eligible |
| 9 | Guday, E., et al. | 2022 | Amhara, | 462 | 57.2 | 12.3 | 98 | WHOQOL-BREF | Cross-sectional | Linear | HKA, MCA and AWA | Nov. 2023 to Decr.2023. | Eligible |
| 10 | Kassie AM, et al. | 2021 | Amhara | 395 | 58.48 | 21.22 | 98.5 | QOLIE-31 | Cross-sectional | Linear | HKA, MCA and AWA | Nov. 2023 to Decr.2023. | Eligible |
| 11 | Mesafint et al. | 2021 | Adiss Ababa | 439 | 61 | 11.6 | 98.2 | WHOQOL-BREF | Cross-sectional | Linear | HKA, MCA and AWA | Nov. 2023 to Decr.2023. | Eligible |
| 12 | Muche, E.A., et al. | 2020 | Amhara | 354 | 19.85 | 6.91 | 99.4 | QOLIE-10 | Cross-sectional | Linear | HKA, MCA and AWA | Nov. 2023 to Decr.2023. | Eligible |
| 13 | Tsigebrhan R et al. | 2021 | Sothern | 237 | 69.7 | 19.3 | 96.3 | QOLIE-10 | Cross-sectional | Linear | HKA, MCA and AWA | Nov. 2023 to Decr.2023. | Eligible |
| 14 | Wudu Yesuf | 2019 | Oromia | 340 | 55.6 | 20.9 | 98.3 | QOLIE-31 | Cross-sectional | Linear | HKA, MCA and AWA | Nov. 2023 to Decr.2023. | Eligible |
| 15 | Hailu, D.S.E. | 2018 | Oromia | 304 | 59.8 | 20 | 96.8 | QOLIE-31 | Cross-sectional | Linear | HKA, MCA and AWA | Nov. 2023 to Decr.2023. | Eligible |
| 16 | Alemu, A., et al. | 2023 | Sothern | 423 | 56.17 | 10.01 | 100 | WHOQOL-BREF | Cross-sectional | Logistic | HKA, MCA and AWA | Nov. 2023 to Decr.2023. | Eligible |

**Note**: AWA; Abere Woretaw Azagew, CKM; Chilot Kassa Mekonnen, HKA; Hailemichael Kindie Abate, MCA; Muluken Chanie Agimas
