# Supplementary figures and images for: Health-related quality of life and its predictors among epilepsy patients in Ethiopia: Systematic review and meta-analysis
Source: PLoS One. 2025 Jun 3;20(6):e0324363. doi: 10.1371/journal.pone.0324363 (PMC12132937; doi:10.1371/journal.pone.0324363)

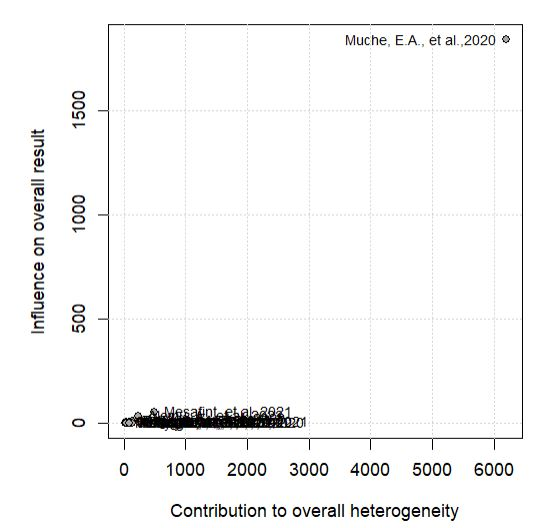

Supplement: S1 Fig — (TIFF) [file pone.0324363.s008.tiff]
